# In Situ Oriented Mn Deficient ZnMn_2_O_4_@C Nanoarchitecture for Durable Rechargeable Aqueous Zinc‐Ion Batteries

**DOI:** 10.1002/advs.202002636

**Published:** 2021-01-04

**Authors:** Saiful Islam, Muhammad Hilmy Alfaruqi, Dimas Yunianto Putro, Sohyun Park, Seokhun Kim, Seulgi Lee, Mohammad Shamsuddin Ahmed, Vinod Mathew, Yang‐Kook Sun, Jang‐Yeon Hwang, Jaekook Kim

**Affiliations:** ^1^ Department of Materials Science and Engineering Chonnam National University Gwangju 500‐757 South Korea; ^2^ Department of Metallurgical Engineering Sumbawa University of Technology Olat Maras Sumbawa West Nusa Tenggara 84371 Indonesia; ^3^ Department of Energy Engineering Hanyang University Seoul 133‐791 Republic of Korea

**Keywords:** aqueous Zn‐ion batteries, in situ grown Mn deficient ZnMn_2_O_4_@C, ZnO‐MnO@C nanocomposite

## Abstract

Manganese (Mn)‐based cathode materials have garnered huge research interest for rechargeable aqueous zinc‐ion batteries (AZIBs) due to the abundance and low cost of manganese and the plentiful advantages of manganese oxides including their different structures, wide range of phases, and various stoichiometries. A novel in situ generated Mn‐deficient ZnMn_2_O_4_@C (Mn‐d‐ZMO@C) nanoarchitecture cathode material from self‐assembly of ZnO‐MnO@C for rechargeable AZIBs is reported. Analytical techniques confirm the porous and crystalline structure of ZnO‐MnO@C and the in situ growth of Mn deficient ZnMn_2_O_4_@C. The Zn/Mn‐d‐ZMO@C cell displays a promising capacity of 194 mAh g^−1^ at a current density of 100 mA g^−1^ with 84% of capacity retained after 2000 cycles (at 3000 mA g^−1^ rate). The improved performance of this cathode originates from in situ orientation, porosity, and carbon coating. Additionally, first‐principles calculations confirm the high electronic conductivity of Mn‐d‐ZMO@C cathode. Importantly, a good capacity retention (86%) is obtained with a year‐old cell (after 150 cycles) at 100 mA g^−1^ current density. This study, therefore, indicates that the in situ grown Mn‐d‐ZMO@C nanoarchitecture cathode is a promising material to prepare a durable AZIB.

## Graphical abstract

A novel battery consisting of zinc anode and Mn deficient ZnMn_2_O_4_@C cathode has been developed by immersing ZnO‐MnO@C electrode in 2 m ZnSO_4_ + 0.2 m MnSO_4_. The in situ formation of a reversible ZnMn_2‐_ *_x_*O_4_@C with Zn_4_(OH)_6_(SO_4_).*x*H_2_O surface layer exhibits extraordinary cycle performance.

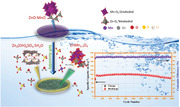

## 1 Introduction

Energy storage is a necessary part of the modern era of ubiquitous high technology. Ecofriendly and high voltage battery technology is required for the safe, long‐term, and convenient operation of advanced electronic devices, such as hand‐held devices, robots, electric vehicles, and medical equipment.^[^ ^1^, ^2^, ^3^, ^4^, ^5^ ^]^ Ion‐based batteries are highly investigated systems for storing energy.^[^ ^6^, ^7^, ^8^, ^9^ ^]^ Among ion‐based batteries, lithium‐ion batteries (LIBs) are widely studied, which are made of highly explosive lithium metal containing with toxic flammable organic electrolyte.^[^ ^10^, ^11^ ^]^ Toxic metal like lead is also used for fabrication of lead‐acid batteries.^[^ ^12^ ^]^ However, considering not only the risk factors for the environment and human health but also cost and lifetime, the future large‐scale applications of LIBs, lead‐acid batteries are hindered. Even the breakthrough research on battery field has been done, still their performances are lower than the satisfactory level for meeting up the ever‐growing energy demand globally. Therefore, finding highly efficient battery technology which should be made of readily available materials and environmentally friendly are still anticipated.

Multivalent ion batteries such as, aluminum, magnesium, nickel, calcium, and zinc‐ion batteries (ZIBs) have garnered significant attention due to the availability and the relatively low price of those metals used.^[^ ^13^, ^14^, ^15^, ^16^, ^17^ ^]^ Also, they are comparatively safe and less toxic than alkali metals. Among the above‐mentioned batteries, ZIBs with aqueous electrolyte have been a target of particular investigation because of their considerable energy density (5851 mAh mL^−1^), substantial redox potential (‐0.76 V vs standard hydrogen electrode) and ecofriendly zinc anode.^[^ ^18^, ^19^, ^20^ ^]^ In addition, it is easy, convenient and does not require any extra‐expensive atmospheric control to manufacture which lead to the low cost.

A suitable cathode material can add significant contribution on overall battery performances. Although, Prussian blue‐based cathode material can maintain a considerable cycle life of the battery, but exhibit very low capacity (nearly ≈ 50 mAh g^−1^).^[^ ^21^, ^22^, ^23^ ^]^ Also, vanadium‐based compounds have been reported as a potentially high capacity cathode material for rechargeable AZIBs.^[^ ^24^, ^25^, ^26^, ^27^, ^28^, ^29^, ^30^, ^31^ ^]^ However, those cathode materials exhibit low voltage compared to that of manganese (Mn)‐based cathode materials.^[^ ^32^ ^]^ The rechargeable AZIB battery system was developed by Kang et al.^[^ ^33^ ^]^ where MnO_2_ was used as a cathode and exhibit a discharge capacity of 210 mAh g^−1^. In the meantime, Pan et al.^[^ ^4^ ^]^ obtained a better result by introducing an electrolyte containing ZnSO_4_ + MnSO_4_ in which MnSO_4_ compensates for the loss of Mn from MnO_2_ cathode. Long‐term stability (capacity retention of 92% over 5000 cycles) and capacity of 285 mAh g^−1^ in respect to the total MnO_2_ are achieved by using a mild aqueous electrolyte of 1 m ZnSO_4_ + 0.1 m MnSO_4_. They suggested that conversion reaction between MnOOH and MnO_2_ played a pivotal part in prolonging the lifetime of the AZIBs. In addition, capacity attenuation and battery failure due to the secondary products (e.g., Mn(OH)_2_, Mn_2_O_3_) formation in the cathode side during cycling also remain great issues.^[^ ^9^, ^34^ ^]^ Furthermore, Alfaruqi et al. also revealed the dissolution of manganese associated with the phase transformation of MnO_2_ in rechargeable AZIB.^[^ ^35^, ^36^, ^37^ ^]^

The formation of ZnMn_2_O_4_ in the cathode due to Zn^2+^ ion insertion at the MnO_2_ cathode was observed for the first time in 2012.^[^ ^33^ ^]^ Afterwards, Chen et al.^[^ ^38^ ^]^ have also introduced ZnMn_2_O_4_ cathode with cation vacancy for an AZIB which exhibits high capacity retention (94%) over 500 cycles (at 500 mA g^−1^ current rate). Simultaneous Zn^2+^ and proton insertion into MnO_2_ in a mild acidic aqueous electrolyte was reported.^[^ ^39^ ^]^ Wei et al. developed a novel in situ formed Zn/MnO_2_ battery where zinc sulphate hydroxide (Zn_4_SO_4_(OH)_6_.5H_2_O) was used as a starting material that initiate an in situ Zn/MnO_2_ cell during the first charging.^[^ ^40^ ^]^ The development and investigation of Mn‐containing cathode materials for AZIBs is still ongoing.^[^ ^41^, ^42^, ^43^ ^]^ It is then worth mentioning that although the Mn‐based cathode are promising over many other transitional metal based cathode, they also possess several drawbacks; and thus they need to be improved.

In this study, we have revealed the in situ growth of ZnMn_2_O_4_@C with Mn deficiency (denoted as Mn‐d‐ZMO@C) from the ZnO‐MnO@C nanocomposite synthesized by a simple solvent dry process for high‐performance rechargeable AZIB. The surface morphology of highly porous and crystalline ZnO‐MnO@C was investigated using SEM and HRTEM. The intrinsic structure and in situ changes of ZnO‐MnO@C were confirmed by X‐ray techniques. Time‐dependent in situ potentiostatic electrochemical impedance spectroscopy (PEIS) revealed the phase alteration of the cathode materials throughout the aging process. Both in situ and ex situ X‐ray diffraction analyses were performed to confirm the formation and reproducibility of Mn‐d‐ZMO@C after assembling the coin cell with ZnO‐MnO@C electrode. The Zn/Mn‐d‐ZMO@C cell exhibited excellent discharge capacity of 219 mAh g^−1^ at 100 mA g^−1^ and cycle stability with discharge capacity of 98 mAh g^−1^ after 2000 cycles with nearly 100% Coulombic efficiency at a high current density of 3000 mA g^−1^. A charged cell can be used after one year and it showed a high capacity retention of 86% over 150 cycles at 100 mA g^−1^ current density. First principle study was used to understand the improved conductivity of Mn deficient electrode. In addition, electrochemical properties of the ZnMn_2_O_4_ and ZnMn_2_O_4_‐MnO samples were also evaluated for comparison purpose.

## 2 Results and Discussion

**Figure 1:**
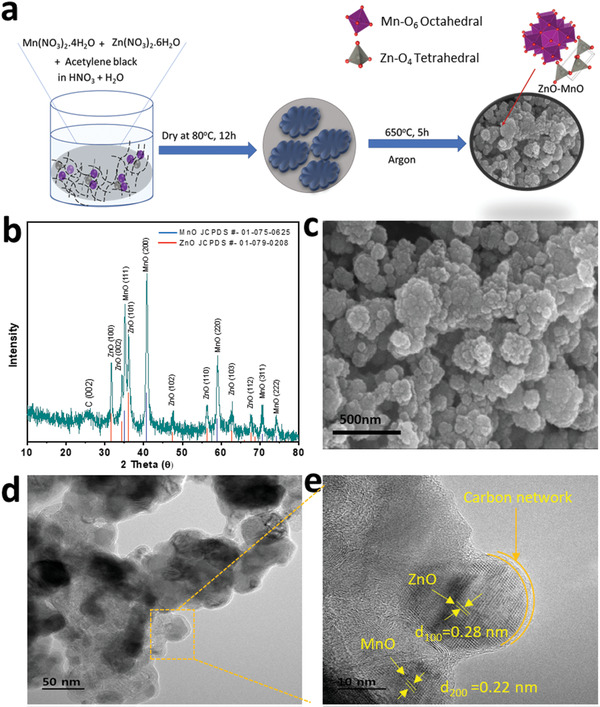
a) Schematic Illustration of synthesis of ZnO‐MnO@C. b) XRD pattern of carbon wrapped ZnO‐MnO. c,d) 500 nm SEM and 50 nm HRTEM image of ZnO‐MnO@C. e) The lattice plane and carbon shell are shown in magnified 10 nm HRTEM image for ZnO‐MnO@C composite materials.

The carbon enfolded ZnO‐MnO is prepared simply by a solvent dry method followed by a calcination at 650 °C in Ar (**Figure** **1**a). The XRD pattern of ZnO‐MnO@C nanocomposite material compared with the standard lines of ZnO and MnO is illustrated in Figure 1b. All X‐ray diffraction peaks in the prepared sample are matched well with the characteristic lines of MnO (JCPDS # 01‐075‐0625), and ZnO (JCPDS # 01‐079‐0208), respectively. The XRD peak near 25° corresponds to graphitic carbon. Here, the ZnO–MnO nanocomposite consists of cubic MnO crystal constructed with Mn‐O_6_ octahedral and hexagonal ZnO crystal built from ZnO_4_ tetrahedral units. Porous spherical‐shaped particles of the prepared sample were observed in the recorded FE‐SEM images (Figure 1c,d, Figure S2a, Supporting Information). A high‐resolution TEM image of ZnO‐MnO@C shows the interface between ZnO and MnO and a clear carbon shell wrapping the former (Figure 1e). This carbon linkage is beneficial to conduct ions, hence increase the electrochemical performance of the electrode.^[^ ^44^, ^45^ ^]^ Lattice plane distances of 0.28 nm corresponds to the ZnO (100) and that of 0.22 nm for MnO (200) (Figure 1e). In order to check the elemental distribution, the mapping of the ZnO‐MnO@C sample was performed. Figure S1a,b (Supporting Information) representing the HAADF and elemental images indicate the uniform elemental distribution of Zn, Mn, C, and O in the sample.

The N_2_ adsorption–desorption study was performed to investigate the porosity features of the ZnO‐MnO@C powder in terms of their surface area, pore size, and pore volume. The resulting isotherm (Figure S2b, Supporting Information) reveals a type‐IV profile with a type‐H1 hysteresis loop, indicating mesoporous particles. The Brunaur‐Emmett‐Teller (BET) surface area was calculated to be 76.62 m^2^ g^−1^_,_ indicating that such high surface area can enhance the electrochemical performance of ZnO‐MnO@C by offering a large interface between the electrolyte and the electrode.^[^ ^46^ ^]^ Further, the pore size distribution plot of the ZnO‐MnO@C powder (inset of Figure S2b, Supporting Information) reveals a pore diameter of 9.55 nm and a pore volume of 0.183 cm^3^ g^−1^. This surface characterization result is therefore consistent with the porous properties observed using SEM analysis.

To study the evolution of the ZnO‐MnO phase at temperatures lower than the synthesis temperature, we studied the samples treated under Ar atmosphere at 400 °C and 500 °C. From the XRD pattern of the low‐temperature calcined powder, it is seen to be well‐indexed with ZnMn_2_O_4_ (JCPDS # 010‐071‐2499) (Figure S3a, Supporting Information). And the powder treated at 500 °C produces ZnMn_2_O_4_ (JCPDS # 01‐077‐0470) with MnO (JCPDS # 01‐075‐0257 indicated with * sign) impurity (Figure S3b, Supporting Information). This indicates that MnO started growing with increasing temperature. As expected, the XRD peak corresponding to the graphitic carbon is observed in all samples. Furthermore, as shown in the SEM images (Figure S3c,d, Supporting Information), the shape of ZnMn_2_O_4_ and ZnMn_2_O_4_‐MnO particles, respectively, are similar to that of the porous spherical ZnO‐MnO@C particle.

Raman analysis was performed for checking the structural information of carbon wrapped ZnMn_2_O_4_, ZnMn_2_O_4_‐MnO, and ZnO‐MnO samples prepared at 400 °C, 500°C and 600 °C, respectively, in Ar. For the initial two samples containing ZnMn_2_O_4_, corresponding Raman bands were observed at 323, 373, and 669 cm^−1^ (Figure S4, Supporting Information).^[^ ^48^ ^]^ The peak around 1146 cm^−1^ observed for the ZnO‐MnO sample corresponds to the ZnO. Because of photochemical irradiation,^[^ ^47^ ^]^ the MnO can be converted to Mn_3_O_4_, thereby reflecting the characteristic peak position around 634 cm^−1^. A couple of strong peaks at 1333 and 1594 cm^−1^ were observed for all three samples (Figure S4, Supporting Information). Here, 1333 cm^−1^ corresponds to the disordered carbon (D‐bond) arising from A_1g_ vibration mode. The Raman peak for graphitic carbon (G‐Bond) located near the 1594 cm^−1^ position is attributed to the E_2g_ vibration mode of ordered carbon. The ratio of *I*_D_/*I*_G_ intensity is close to 1, demonstrating a sufficient amount of graphitization which is beneficial for the smooth conduction of electrons.^[^ ^49^ ^]^ Further characterizations were focused mainly on ZnO‐MnO@C since it is the target sample of the present study.

In order to check elements and the oxidation state of the manganese in ZnO‐MnO@C, X‐ray photoelectron spectroscopy was performed. The survey scan for ZnO‐MnO@C confirms the position of notable elements (Zn, Mn, O) and is provided in the Figure S5a (Supporting Information). The binding energies of 641.0 and 653.37 eV can be ascribed to Mn 2p_1/2_ and Mn 2p_3/2_ respectively (Figure S5b, Supporting Information) which indicates the presence of manganese as Mn^2+^ in MnO. Further, the peaks located at 1021 and 1044 eV are characteristic of Zn 2p_3/2_ and Zn 2p_1/2_, respectively (Figure S5c, Supporting Information).

**Figure 2:**
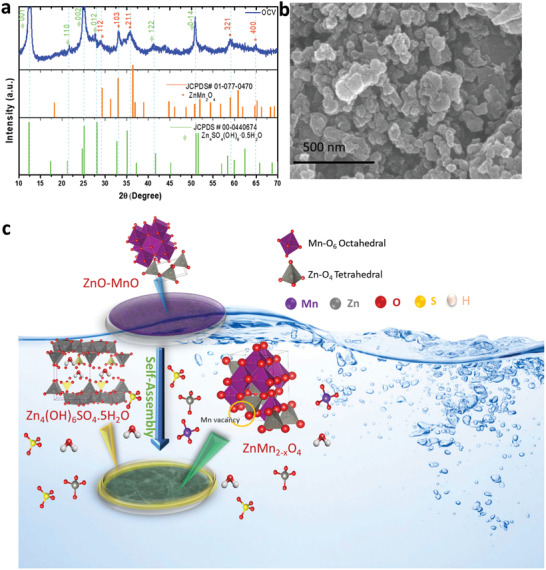
a) Ex situ XRD pattern and b) corresponding SEM image of aged ZnO‐MnO@C electrode, and c) schematic illustration of in situ formation of Mn deficient ZnMn_2_O_4_@C.

The in situ cell of Zn/Mn‐d‐ZMO@C was obtained while aging the assembled coin cell for 24 h where zinc was used as an anode, the ZnO‐MnO@C electrode in the counterpart and an aqueous solution of 2 m ZnSO_4_ and 0.2 m MnSO_4_ was used as an electrolyte. The XRD of the electrode was checked after recovering the ZnO‐MnO@C electrode from 24 h aged coin cell. **Figure** **2**a shows that the XRD patterns are well matched with ZnMn_2_O_4_ (JCPDS # 01‐077‐0470) and Zn_4_(OH)_6_(SO_4_).0.5 H_2_O (JCPDS # 00‐044‐0674) (ZBS). The peak near 12.5°, 25.0°, and 50.80° corresponds to the plan (001), (002), and (0‐14) of (Zn_4_(OH)_6_(SO_4_). H_2_O) and XRD peak at 29.03°, 33.11, 35.52°, 59.06°, 65.0° for the plane (112), (103), (211), (321), and (400) of ZnMn_2_O_4_. The ZBS can be formed depending on the pH of the electrolyte.^[^ ^50^ ^]^ The pH of the electrolyte of the aged coin cell was around 4.5 which is favorable for ZBS stability. Interestingly, highly reversible Mn‐d‐ZMO@C was formed simultaneously, which acts as a cathode material and initiates an in situ Zn/Mn‐d‐ZMO@C battery. Since the cathode surface was covered by ZBS precipitate, to obtain clear XRD pattern of the newly formed ZMO phase, we have then performed ex situ XRD analysis of the aged ZnO‐MnO@C electrode after washing with pH ≈ 3 water.^[^ ^50^ ^]^ As depicted in Figure S6 (Supporting Information), the XRD pattern is well indexed with the ZnMn_2_O_4_ (JCPDS # 01‐077‐0470). It is highly like that ZnO reacted with MnO produces Mn‐d‐ZMO@C in the presence of the electrolyte.^[^ ^51^ ^]^ Mn^2+^ may also dissolve from ZnO‐MnO@C into the electrolyte while the formation of ZnMn_2_O_4_ with Mn vacancy occurred. A similar kind of phenomenon was investigated by the Wang et al., where they performed electrodeposition to oxidize Mn^2+^ to Mn^4+^.^[^ ^39^ ^]^ Furthermore, a Mn^2+^/MnO_2_ battery was developed by the Chen et al. In their study, soluble Mn^2+^ and solid MnO_2_ was obtained while 4 m MnSO_4_ was used as an electrolyte.^52^ Also, Huang et al.^[^ ^57^ ^]^ recently reported the participation of Mn^2+^ in a Zn/MnO_2_ aqueous batteries, where they calculated the Gibbs free energy of ZnMn_2_O_4_ in the Zn‐Mn‐H_2_O system was ‐293.439 kcal mol^−1^ indicating that the formation of ZnMn_2_O_4_ is more feasible in our Zn/ZnO‐MnO@C aqueous system. Moreover, ZBS reacts with the dissolved Mn^2+^ in the electrolyte produces more Mn‐d‐ZMO@C.

We further attempted to measure the chemical composition of the cathode. To exclude additional contribution of Zn content from Zn(OH)_2_SO_4_.H_2_O, we have treated the sample using mildly acidic water (pH 3) to remove the precipitate prior to the measurements. It was found that a mildly acidic water could effectively remove the precipitate.^[^ ^50^ ^]^ ICP and XPS analyses of the treated‐electrode suggest that the presence of Mn vacancy in the in situ growth Mn‐d‐ZMO@C, which will be beneficial for electrode applications.^[^ ^38^ ^]^ Precisely, the binding energy of around 642 and 653.5 eV observed for Mn 2p_3/2_ and Mn 2p_1/2_, which indicating peak shifting to higher energy region resulted after aging process (Figure S7, Supporting Information). It also suggests the oxidation of manganese occurred during aging process. In addition, the atomic ratio of Zn to Mn was measured to be ≈1.72, depicting the chemical formula of the Mn deficient ZnMn_2_O_4_ is approximately ZnMn_1.72_O_4_; and thus the theoretical capacity of the sample can be calculated to be 239 mAh g^−1^. Moreover, the presence of Zn(OH)_2_SO_4_ H_2_O is easily distinguishable to the naked eye as a white layer covering on the electrode surface. Hence, during cleaning of the recovered electrode using mild acid, the white precipitative layer peeling off from the electrode is visible. Furthermore, manual scraping of the recovered electrode surface and the collection of the internal active material ensures that the contribution of Zn(OH)_2_SO_4_ H_2_O becomes insignificant during ex situ measurements. Finally, we compared the ICP results of the prepared powder and the aged electrode sample to clearly understand the manganese deficiency in the latter.

The morphological evolution of the cathode was also studied. It can be seen from the SEM image that the shape of the electrode remains unchanged after aging the assembled coin cell (Figure 2b). Further HRTEM analysis was performed to identify the corresponding lattice planes for ZnMn_2_O_4_. As illustrated in Figure S8 (Supporting Information) , the lattice planes 111, 103, and 211 are for zinc inserted spinel ZnMn_2_O_4_.^[^ ^54^ ^]^ Elemental mapping (Figure S8c, Supporting Information) confirmed the elemental distribution of Zn, Mn, S, and O and the growth of Zn_4_(OH)_6_(SO_4_).*x*H_2_O layer. This analysis revealed the formation of Mn‐d‐ZMO@C after aging the electrode. As illustrated in Figure 2C, our proposed reaction mechanism for the in situ formation of Mn deficient ZnMn_2_O_4_ is as follows:

(1) $$\begin{array}{ccc} & & 4Zn^{2+}+{\mathrm{SO}}_{4}^{2-}+5H_{2}O\\ & & \;+\;6OH^{-}\overset{2M\;\mathrm{ZnS}O_{4}+0.2M\;\mathrm{MnS}O_{4}}{\to }\;Zn_{4}{\left(\mathrm{OH}\right)}_{6}SO_{4}.5H_{2}O \end{array}$$

(2) $$\begin{array}{ccc} & & 2\mathrm{MnO}+\mathrm{ZnO}+H_{2}O\overset{2M\;\mathrm{ZnS}O_{4}+0.2M\;\mathrm{MnS}O_{4}}{\to }\;\mathrm{ZnM}n_{2-x}O_{4}\\ & & \;+\;xMn^{2+}+H_{2} \end{array}$$

(3) $$\begin{array}{ccc} & & 6Mn^{2+}+4Zn_{4}{\left(\mathrm{OH}\right)}_{6}SO_{4}.H_{2}O\to 3\mathrm{ZnM}n_{2-x}O_{4}+3xMn^{2+}\\ & & \;+\;4{\mathrm{SO}}_{4}^{2-}+32H_{2}O+13Zn^{2+}+6e^{-} \end{array}$$

To obtain more insight into phase transformation of the electrode, the as‐prepared electrode was immersed in the ZnSO_4_ + MnSO_4_ electrolyte for 12 and 24 h without Zn anode. An ex situ XRD characterization for the electrode was performed after the immersion (Figure S9, Supporting Information). It can be observed that the ZnO‐MnO@C electrode was converted after immersing in the electrolyte. Compared to the in situ aged electrode which shows prominent phase transformation, for the immersed electrode, the new phases of ZnSO_4_.3Zn (OH)_2_.4H_2_O and spinel ZMO were continuously formed; however, some portion of the ZnO.MnO phase remained unreacted. This can be associated with the potential difference arises in the assembled coin cell. Nevertheless, it can be inferred that ex situ XRD analysis after immersing the electrode without Zn anode indicates phase transformation of the cathode was thermodynamically favored.

XANES analysis was performed to investigate the oxidation state of the Mn in carbon enfolded ZnMn_2_O_4_, ZnMn_2_O_4_‐MnO, and ZnO‐MnO and in situ formed Mn‐d‐ZMO@C with respect to the standard materials MnO, Mn_2_O_3,_ and MnO_2_. Figure S10a (Supporting Information) shows that the XANES curve for ZnO‐MnO and reference material MnO are close enough to overlap. Similarly, the curve for ZnMn_2_O_4_ and ZnMn_2_O_4_‐MnO are almost superimposed on that of the Mn_2_O_3_. Therefore, the oxidation state of Mn in ZnMn_2_O_4_, ZnMn_2_O_4_‐MnO is close to 3+ with respect to the reference materials Mn_2_O_3_. As expected, Mn^2+^ was observed for the ZnO‐MnO@C sample. It can be seen that the Mn^3+^ in ZnMn_2_O_4_ reduced with the increasing temperature. At 500 °C the oxidation state of Mn in ZnMn_2_O_4_ started to reduce, and it reached Mn^2+^ at 650 °C which is in agreement with the oxidation state of Mn in the molecular formula of ZnO‐MnO confirmed from the XRD pattern. Interestingly, the Mn in ZnO‐MnO oxidized back to Mn^3+^ after aging the ZnO‐MnO electrode in a zinc cell containing ZnSO_4_ and MnSO_4_ aqueous solution as an electrolyte. In agreement with the XANES analysis, the formation of ZnMn_2_O_4_ was confirmed from the XRD pattern of the recovered electrode. A clearer picture can be seen when comparing Mn valance versus energy in Figure S10b (Supporting Information). We also performed voltage change measurement of the Zn/ZnO‐MnO cell during aging process. Voltage change analysis suggests that the cell underwent an increased potential from 1.27 to 1.34 V and is stabilized after 24 h which further confirms the oxidation of Mn^2+^ to Mn^3+^ happened during the aging process (Figure S11, Supporting Information). This observation also supports the in situ formation of ZnMn_2_O_4_.

**Figure 3:**
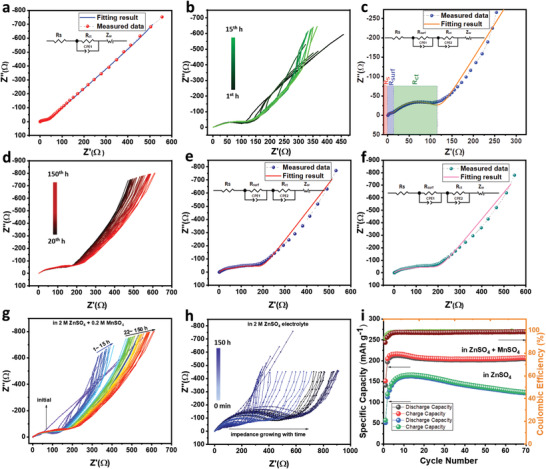
In situ EIS profile of ZnO‐MnO@C at different time. a) Impedance for instantly prepared coin cell with a fitting curve, b) impedance curve recorded from 1 to 15 h, c) fitted curve with an equivalent circuit for the impedance after 7 h aging, d) in situ EIS profile within 20 to 150 h. e) Nyquist plot at 30^th^ h and f) 90^th^ h, comparative EIS of the ZnO‐MnO@C electrode in different electrolytes g) in 2 m ZnSO_4_ + 0.2 m MnSO_4_, h) in 2 m ZnSO_4_, i) cycle performance in 2 m ZnSO_4_ with and without 0.2 m MnSO_4_ electrolyte at 300 mA g^−1^ current rate.

The aging process further studied using (PEIS). We have taken PEIS immediately after assembling the coin cell and recorded at different times. As depicted in **Figure** **3**a, the overall impedance of the instantly prepared cell is around 36 Ω only. In the equivalent circuit, *R*_ct_, *R*_s_, CPE, and *Z*_w_ represent charge transfer resistance, the resistance of electrolyte, the constant phase element, and Warburg impedance, respectively. It can be observed from the Figure 3b that resistance growing with the increasing time. As represented in the Figure 3c, a new semicircle appeared after 7 h, which is indicated by surface resistance (*R*_surf_). This is due to the formation of Zn_4_(OH)_6_SO_4_.*x*H_2_O (ZBS). Interestingly, a similar semicircle observed from 20th to 150th h and all impedance curves are overlapped on each other until 150 h. Indicating a stable phase formed after 24 h (Figure 3d). The fitted curve after 30 and 90th h showed a similar pattern which further ensured the formation of stable phase (Figure 3e,f). This phase is due to the formation of Mn‐d‐ZMO@C and Zn_4_(OH)_6_SO_4_.*x*H_2_O. In situ PEIS study is consistent with the ex situ XRD analysis.

For comparison, we have investigated PEIS of the ZnO‐MnO@C nanocomposite in ZnSO_4_ electrolyte with and without MnSO_4_ additives. As displayed in the Figure 3g, the cell containing MnSO_4_ in ZnSO_4_ electrolyte shows that impedance stabilizes after 24 h. Whereas more resistance has grown continuously in the absence of MnSO_4_ additives (Figure 3h). This result reflected in the electrochemical performances. While considering the electrochemical performance of the cell consisting of aqueous 2 m ZnSO_4_ electrolyte with and without additive (0.2 m MnSO_4_) (Figure 3i). From the charge–discharge pattern (Figure S12a, Supporting Information) it can be clearly seen that in 2 m ZnSO_4_ electrolyte with additive, the cell exhibits lower polarization than that of the cell operated in 2 m ZnSO_4_ electrolyte. It can be observed from the Figure S12a (Supporting Information) that the initial charge capacity for the ZnO‐MnO@C electrode in an aqueous solution of ZnSO_4_ + MnSO_4_ is 151 mAh g^−1^ at 300 mA g^−1^ current density, whereas it is only 57 mAh g^−1^ in ZnSO_4_ electrolyte. Similarly, a high discharge capacity of 141 mAh g^−1^ was obtained in the presence of MnSO_4_ additives. In both cases, the initial activation process was observed while checking cycle ability (Figure 3i). After 70 cycles the cell with additive containing electrolyte registered 204 mAh g^−1^ discharge capacity at 300 mA g^−1^ rate, meaning that it retains 96% discharge capacity with respect to the highest discharge capacity of 212 mAh g^−1^ obtained at the eighth cycle. Without MnSO_4_, it retains only 74% of discharge capacity after 70 cycles (122 mAh g^−1^ capacity at the 70th cycle versus 163 mAh g^−1^ at the 12th cycle).

**Figure 4:**
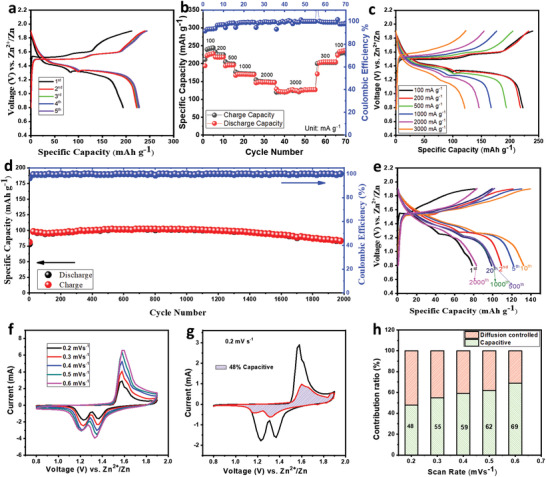
Electrochemical performance of the ZnO‐MnO@C electrode. a) Charge–discharge profile at 100 mA g^−1^ current density between 0.8 and 1.9 V. b,c) Rate capability at different current rate with corresponding ECD pattern and d,e) Cycle performance at 1000 and 3000 mA g^−1^ with ECD pattern respectively. f) CV profile at 0.2, 0.3, 0.4, 0.6 mV s^−1^ scan rate. g) Capacitive portion embedded in the CV curve. h) Capacitive and diffusion contribution versus scan rate curve.

Cyclic voltammetry analysis was performed and is discussed for analysis reaction mechanism of Zn/ZnO‐MnO@C cell. From the CV profile, the oxidation peak at 1.58 V appeared at the very first cycle while the cell operated at a scan rate of 0.2 mV s^−1^. As shown in Figure S12b (Supporting Information), two distinct cathodic peaks were observed at 1.22 and 1.39 V. An increase of peak intensity in the subsequent cycle showed the activation process which agrees with the characteristics of charge/discharge. A similar phenomenon was observed in a previously reported article.^[^ ^54^ ^]^ Electrochemical charge–discharge was conducted within the potential window of 0.8 to 1.9 V. As shown in **Figure** **4**a, when charged to 1.9 V, the in situ Zn/Mn‐d‐ZMO@C cell with 2 m ZnSO_4_ + 0.2 m MnSO_4_ electrolyte registered 212 mAh g^−1^ capacity and a discharge capacity of 194 mAh g^−1^ at a current density of 100 mA g^−1^. At the fifth cycle, the cell registered a high capacity of 227 mAh g^−1^. When checking rate capability at different current rates, sacrificial ZnO‐MnO@C cathode materials display very promising performance. Figure 4b shows an average discharge capacity of 219, 218, 196, 169.58, 148, 132 mAh g^−1^ observed at 100, 200, 500, 1000, 2000, and 3000 mA g^−1^ current rate, respectively. After cycling at different current density, the electrode was able to register discharge capacity of 201 and 233 mAh g^−1^ when it was operated at 300 and 100 mA g^−1^ rate, respectively. The electrochemical charge–discharge curve at the various current rates is shown in Figure 4c. As shown in Figure 4d,e, long cycle ability has been checked at two different current rates, with the first 10 cycles at 1000 mA g^−1^ and the remainder of the cycles at 3000 mA g^−1^ current density. For the first cycle, the Zn/Mn‐d‐ZMO@C cell registered a discharge capacity of 78 and 81 mAh g^−1^ charge capacity at a very high current density of 1000 mA g^−1^. A high capacity of 132 mAh g^−1^ was recorded after 10 cycles. The preliminary rise in capacity is due to the stimulation process, which agrees with previously reported articles.^[^ ^53^ ^]^ When the cell is cycled at 3000 mA g^−1^ current rate, 92.5 mAh g^−1^ discharge capacity was observed at the 11th cycle. It reached 98 mAh g^−1^ capacity at the 14th cycle and sustained a high number of cycles with good capacity retention. After 2000 cycles, it recovered 82.7 mAh g^−1^ discharge capacity, i.e., it retained 84% capacity (with respect to the highest capacity of 98 mAh g^−1^). The charge–discharge profile at different cycles as shown in Figure 4d shows a similar charge‐discharge pattern throughout the all cycles. It can be concluded that the in situ formed Mn‐d‐ZMO@C cathode is highly reversible.

To understand the superiority of the in situ generated Mn‐d‐ZMO@C electrode prepared in the present study, the electrochemical performances of ZnMn_2_O_4_@C, ZnMn_2_O_4_/MnO@C and ZnO‐MnO@C electrodes in MnSO_4_ additive containing ZnSO_4_ electrolyte, respectively, were measured and compared (Figure S13, Supporting Information). Electrochemical cyclic voltammetry was performed within the potential range 0.8 to 1.9 V at a scan rate of 0.2 mV s^−1^. Figure S13a (Supporting Information) represents the anodic peak near 1.59 V and the cathodic peaks at 1.39 and 1.15 V were observed for ZnMn_2_O_4_@C. Similar results were observed for ZMO with MnO impurity (Figure S13b, Supporting Information). Both observations are consistent with the CV profile of in situ cell developed from the ZnO‐MnO@C nanostructure materials. Electrochemical charge–discharge properties of the ZnMn_2_O_4_@C and ZnMn_2_O_4_/MnO@C were also evaluated at a 500 mA g^−1^ rate. It can be observed that in situ generated cathode from ZnO‐MnO exhibited less polarization than those of ZnMn_2_O_4_ and ZnMn_2_O_4_‐MnO electrodes (Figure S13c, Supporting Information). When comparing their cycle ability performances, pure ZnMn_2_O_4_ registered only 20 mAh g^−1^ discharge capacity at 500 mA g^−1^ current density, whereas they were 56 and 118 mAh g^−1^ for ZnMn_2_O_4_‐MnO and ZnO‐MnO, respectively. Moreover, the Mn‐d‐ZMO@C cathode delivered stable long cycle life (Figure S13d, Supporting Information). It can be concluded that the incorporation of MnO enhanced the electrochemical performance of the ZMO electrode significantly. In addition, the electrochemical cycle performance of the Zn/ZnO‐MnO@C electrode was investigated after a long time (1 year). The assembled cell was kept after charging up to 1.9 V and discharged after 1 year. At the 150^th^ cycle, this 1 year old cell can still deliver a discharge capacity of 60 mAh g^−1^ with no substantial capacity fading (nearly 86% capacity retention) at 100 mA g^−1^ current rate (Figure S14, Supporting Information).

The cyclic voltammetry (CV) analysis of the Zn/ZnO‐MnO@C cell was performed to study the further reaction mechanism and chemical kinetics. Investigation of chemical kinetic of the cell, cyclovoltammetry was employed at different scan rates, namely (0.2, 0.3, 0.4, 0.5, and 0.6 mV s^−1^) and displayed in Figure 4f. The following equation is used to measure the chemical kinetics of the cell:^[^ ^55^ ^]^

(4) $$i\left(V\right)=k_{1}v+k_{2}v^{1/2}$$

Here *i* is the current response at a static potential (*V*), *v* is the scan rate, *k*_1_ and *k*_2_ are constants use to determine the capacitive and diffusion‐limited behavior, respectively. The calculated capacitive behavior of the electrode was 48% at 0.2 mV s^−1^ rate and is shown by the shaded area in Figure 4g. From the bar diagram (Figure 4h) and the Figure S15a–d (Supporting Information) it can be seen that the capacitive contribution is 55%, 59%, 62%, and 69% at 0.3, 0.4, 0.5, and 0.6 mV s^−1^ sweep rates, respectively.

**Figure 5:**
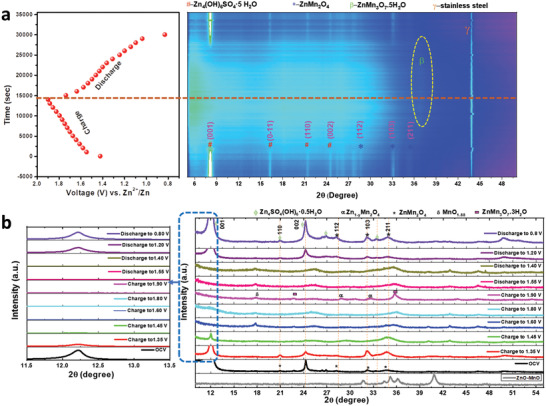
a) In situ XRD of ZnO‐MnO@C with corresponding charge–discharge profile. b) Ex situ XRD of Zn/ZnO‐MnO@C at different state of charge–discharge.

In situ XRD analysis was performed to investigate the structural change during electrochemical measurement. From **Figure** **5**a the in situ formation of ZnMn_2_O_4_ (indicated by the * sign) can be clearly seen, along with Zn_4_(OH)_6_SO_4_.5H_2_O (specified with the # sign) and an unavoidable peak for stainless steel (identified with the *γ* sign). More water content was identified for the in situ situation but is reduced while checking the XRD of the dried electrode. A total of 137 XRD measurements were taken during charge–discharge. When the electrode was subjected to charge, the peak for Zn_4_(OH)_6_SO_4_.5H_2_O (ZBS) started to weaken due to the dissolution of ZBS because of the increasing acidity of the electrolyte. While the electrode charged completely, the pH value was reduced to around 3 and ZBS precipitate can be easily dissolved. Previous report also suggested after charging, the XRD peak for ZBS was abolished completely.^[^ ^56^ ^]^ The plane 103 for ZnMn_2_O_4_ also almost disappeared at the charged state. Remarkable peak shifting of plane 211 is observed from the Figure 5a. The appearance of all peaks after discharge indicates the reversibility of the in situ grown electrode. For more details, we have studied the ex situ XRD at different states of charge–discharge. As depicted in Figure 5b, the planes 001 and 002 for ZBS were abolished after a full charge. At the beginning of charging, the peak for ZBS became weaker because of the dissolution of the ZBS precipitate. This allows clearer identification of ZMO. At first, a very strong peak was observed at 2*θ* angles from 11.5° to 13.5°. The plane 001 corresponding to ZBS disappeared after charging and reformed after discharging. Similar phenomena were observed for the 002 plane and the rest of the planes for ZBS. 112, 103 and 211 planes of ZMO were considered for further analysis. From Figure 5b it can be observed that the peak near 28° and 33° are shifted slightly to the right after full charging, which corresponds to the formation of Zn_1‐_ *_y_*Mn_2_O_4_ (identified by an *α* sign), whereas the diffraction peak for 211 plane shifted substantially. New peaks near 22° and 35.76° are identified as ZnMn_3_O_7_.3H_2_O (PDF # 15‐0807).^[^ ^57^ ^]^ While some studies showed that during charging ZBS dissolution occurred due to the change of the electrolyte pH,^[^ ^4^, ^14^, ^58^, ^59^, ^60^ ^]^ we also observed the formation of ZnMn_3_O_7_.3H_2_O, which is in agreement to the reports by Zhao et al. and Huang et al.^[^ ^40^, ^57^ ^]^ It was found that Mn^2+^ additive enhanced the deformation of ZBS by producing ZnMn_3_O_7_ which could also act as a Zn host and influenced the initial capacity significantly (Figure 3i). Thus, the trend of the present XRD results seem to agree with this conversion reaction in addition to the usual Zn‐deintercalation reaction upon charging. Furthermore, the emergence of a new XRD peak close to 18° corresponding to manganese oxide (MnO_1.88_, JCPDS # 00‐005‐0673) is observed. This points to the feasibility of MnO_2_ getting deposited on to the electrode surface via the oxidation of Mn^2+^ additive ions in the electrolyte during the initial charge cycling. The overall XRD results, thus conclude that the deintercalation of Zn leading to Mn oxidation in the bulk occurs upon initial charging. Besides intercalation, a major role is suspected to be played mainly by the Mn^2+^ additive ions in the reaction mechanism. In specific, a portion of the Mn^2+^ ions, mostly from the additive, undergoes a conversion reaction with ZBS on the electrode surface to produce ZnMn_3_O_7_.3H_2_O. On further charging, the remnant Mn^2+^ ions electrodeposit as MnO*_x_* via oxidation. Moreover, Mn^2+^ also affects the Coulombic efficiency of the cell while cycling at a low current density due to the formation of ZnMn_3_O_7_ and MnO_1.88_ at the initial stage (Figure 4b). In addition, parasitic reactions such as hydrogen evolution at the initial cycle may also responsible for the low Coulombic efficiency.^[^ ^61^ ^]^

When the cell is discharged up to 0.8 V, the 112, 103, and 211 planes were recovered completely; this proved the structural reversibility of the in situ electrode. For further clarification, the XRD pattern of the electrode was also recorded after 10 cycles and compared with the pristine electrode (Figure S16, Supporting Information). This peak shifts due to the existence of less water content in the ex situ samples.^[^ ^50^ ^]^ The shifted peaks originated from the variation of water content within the structure and this was also observed in the previous study, while the new peak near 17° and 18° can be assigned as Zn_4_(OH)_6_SO_4_.0.5H_2_O and ZnMn_2_O_4_, respectively, which became more prominent after cycling (Figure S16, Supporting Information). It is worth noting that the overlapped diffraction peaks make it more difficult to analyze the phases; however, we can clearly observe ZnMn_2_O_4_ phase of the acid‐washed electrode (Figure S6, Supporting Information). In addition, we have also performed ex situ XRD and SEM analyses of the in situ formed electrode after 20 cycles and the results are depicted in Figure S17 (Supporting Information). Strong ZnMn_2_O_4_ (JCPDS # 010‐077‐0470) along with ZBS (JCPDS # 039–0690) characteristics can be clearly observed after 20 cycles from XRD patterns. Notably, SEM image indicates the spherical shape of the particle preserved after cycling. These observations suggest the high reversibility and stability of the cathode during consecutive cycling.

The relatively lower Coulombic efficiency during the initial few cycles at low current density (≈100 mA g^−1^) is worth noting. The present study clarifies that the reaction mechanism in the Mn‐d‐ZMO@C electrode can be explained by an intercalation and two conversion processes, respectively. The deintercalation, in general, is believed to be dominant at low current densities and, in that case, it is feasible that the deintercalated Zn^2+^ions are not fully inserted back into the electrode bulk during the discharge reaction either due to the presence of irreversible sites or because of some hindrance to the motion of the intercalating Zn^2+^ ions. The low current density can motivate the formation of a relatively thick ZBS layer on the electrode surface during discharge cycling. Thus, it can be possible that the difficulty in penetration of Zn^2+^ ions through this thick layer formed at the electrode/electrolyte interface may not allow the full intercalation of the deintercalated Zn^2+^ ions. Also, this irreversibility can be related to some unusual pH variation that can hinder the reversibility of either of the conversion reactions when cycled at low current densities.

Ex situ synchrotron XAS analysis was performed at a different state of charge–discharge to study the change of manganese oxidation state of the ZnO‐MnO@C electrode. The Mn K‐edge spectrum of the aged electrode close to that of Mn_2_O_3_ reference, representing that the oxidation of manganese during aging process (Figure S18, Supporting Information). While charging, the energy of Mn K‐edge moves towards higher energy. At the fully charged state (1.90 V) the Mn K‐edge of the electrode (purple colored curve) is nearly superimposed on that of the MnO_2_ reference (black colored curve). This is highly likely due to the presence of ZnMn_3_O_7_ and Mn vacancy in the spinel phase electrode. During discharge, the reverse phenomena occurred (Figure S19, Supporting Information). At the depth of discharge of 0.8 V, a very similar Mn K‐edge spectrum was obtained as that observed for the aged electrode. That means Mn^3+^ is regained after fully discharged. This result supported both the in situ and ex situ XRD analyses. It is also worth mentioning that in the discharge reaction Mn^2+^ exists in the electrolyte solution. As displayed in Figure S18 (Supporting Information), the Mn K‐edge for the first and second charges were comparable, showing the reversibility of the electrode reaction. Moreover, we have performed ex situ XPS analysis during cycling. As shown in Figure S20 (Supporting Information), Mn2p_3/2_ and Mn2p_1/2_ peaks move to higher energy after the aging process. Peak near the 641.5 eV corresponds to Mn^3+^. It can be seen that the binding energy increases further while the electrode charged to 1.9 V and majority of the Mn2p_3/2_ peak near the 642.2 eV indicating the oxidation of Mn and similar peak shifting observed for Mn2p_1/2_ peak.^[^ ^62^ ^]^ The ex situ XPS results were closely resemblance to the XANES analyses. Overall, the repetition of nearly Mn^4+^/Mn^3+^ redox couple was observed during charge/discharge.

**Figure 6:**
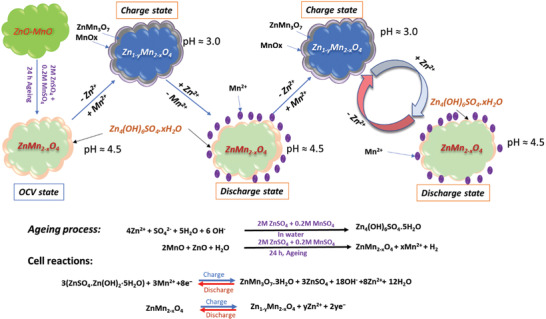
Schematic illustration for reaction mechanism of the in situ formed Zn/Mn‐d‐ZMO@C battery.

In the light of above discussion, at first ZnO‐MnO@C undergoes a “self‐oxidation” in an aqueous solution of ZnSO_4_ + MnSO_4_ electrolyte. Further, it converted into spinel ZnMn_2_O_4_ phase and the of Zn_4_(OH)_6_.SO_4_.5H_2_O (ZBS) precipitate was formed on the surface of the electrode materials. Further, ZBS reacts with Mn^2+^ to produce more ZMO, which is consistent with the previous reports.^[^ ^38^, ^40^ ^]^ When being charged, the oxidation of the Mn occurred. Specifically, Zn_1‐y_Mn_2‐x_O_4_ and ZnMn_3_O_7._3H_2_O were formed after charging. It is to be noted that at charged state, the pH of the electrolyte was reduced to 3.0 from 4.5, while in the fully discharged state, ZnMn_2_O_4_ reformed, whereas electrolyte pH was found to be 4.5. As also illustrated in the **Figure** **6**, the proposed electrochemical charge‐discharge mechanism of the in situ cell is as follows:

Charge:

(5) $$\mathrm{Cathode}:\mathrm{ZnM}n_{2-x}O_{4}↔Zn_{1-y}Mn_{2-x}O_{4}+yZn^{2+}+2ye$$

(6) $$\begin{array}{ccc} & & 3\left(\mathrm{ZnS}O_{4}.\mathrm{Zn}{\left(\mathrm{OH}\right)}_{2}\cdot 5H_{2}O\right)+3Mn^{2+}+8e^{-}↔\mathrm{ZnM}n_{3}O_{7}.3H_{2}O\\ & & \;+3\mathrm{ZnS}O_{4}+18OH^{-}+8Zn^{2+}+12H_{2}O \end{array}$$

(7) $$M{n^{2+}}_{\left(\mathrm{ad}\right)}+xH_{2}O↔\mathrm{Mn}O_{x\left(\mathrm{dep}\right)}+x\;2H^{+}+2e^{-}$$

(8) $$\mathrm{Anode}:Zn^{2+}+2e^{-}↔\mathrm{Zn}$$

Discharge:

(9) $$\mathrm{Cathode}:\;Zn_{1-y}Mn_{2-x}O_{4}+{\mathrm{Zn}}_{y}^{2+}+2ye^{-}↔\mathrm{ZnM}n_{2-x}O_{4}$$

(10) $$\begin{array}{ccc} & & \mathrm{ZnM}n_{3}O_{7}.3H_{2}O+3\mathrm{ZnS}O_{4}+18OH^{-}+8Zn^{2+}+12H_{2}O\\ & & \;↔3\left(\mathrm{ZnS}O_{4}.\mathrm{Zn}{\left(\mathrm{OH}\right)}_{2}\cdot 5H_{2}O\right)+3Mn^{2+}+8e^{-} \end{array}$$

(11) $$\mathrm{Anode}:\;\mathrm{Zn}↔Zn^{2+}+2e^{-}$$

The reaction mechanism of manganese oxide cathodes in the Zn//MnO_2_ system is hugely debated at present.^[^ ^4^, ^14^, ^40^, ^57^, ^58^, ^59^, ^60^ ^]^ Briefly, different type of reactions was proposed to describe the mechanism in manganese oxide cathodes for AZIBs. Initially, pure Zn‐intercalation was floated as the plausible reaction mechanism.^[^ ^33^, ^53^ ^]^ Thereafter, the possibility of a dual Zn^2+^/H^+^ intercalation regulation was put forward.^[^ ^39^, ^63^ ^]^ However, controversies still exist in assigning the specific ion related to the pathway of the main driving reaction. Meanwhile, conversion reactions based on a proton‐transfer process and a phase transfer process, respectively, were also deemed possible to govern the electrochemical regulations in manganese oxides of AZIBs.^[^ ^4^ ^]^Also, the possibility of mixed intercalation‐cum‐chemical conversion reactions were proposed.^[^ ^64^ ^]^ Very recently, a manganese deposition/dissolution process that challenges the views of conventional intercalation was presented.^[^ ^65^ ^]^ In this regard, we propose that the reaction mechanism in the present Mn‐d‐ZMO@C cathode can be described by a combined intercalation‐conversion‐deposition reaction in an Mn^2+^‐containing ZnSO_4_ aqueous electrolyte medium.

Further in situ PEIS analysis was conducted for investigating the change of resistance and phase formation during cycling. From Figure S21a (Supporting Information) it can be elucidated that during first charge, over all resistant (*R*) decreases from around 178 to 143 Ω at 1.35 V. This is maybe due to the gradual deformation of more ZBS which was well observed in ex situ XRD analysis. Interestingly, resistant further reduced in the subsequent charge state and only 18 Ω observed when it was charged to 1.90 V. This result suggest that the deformation of Mn‐d‐ZMO@C occurs during charging. When it is discharged, the *R* value increases dramatically, and this is due to the reversible formation of the ZnMn_2_O_4_ and ZBS layer (Figure S21b, Supporting Information). Similar phenomena were observed in the subsequent cycles (Figure S21c,d, Supporting Information). Based on electrochemical results, the in situ grown Mn‐d‐ZMO@C shows superior electrochemical performance. In addition, ICP analyses suggest the dissolution of Mn, which leads to the deficient amount of Mn in the in situ grown sample. With the Mn vacancy defect, it is expected that the material shows an improved electrochemical performance.

**Figure 7:**
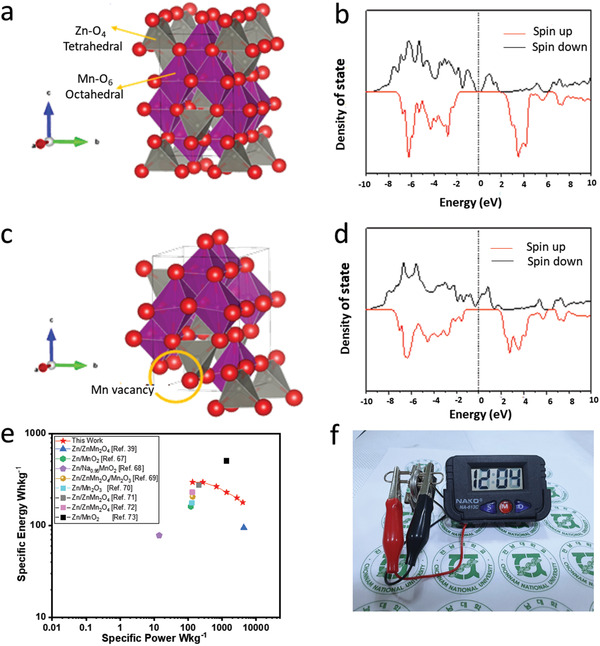
a) Illustration of crystallographic structures of ZnMn_2_O_4_. b) Total density of states (DoS) of ZnMn_2_O_4_ c) crystallographic structures of ZnMn_2_O_4_ with Mn vacancy defect. d) Total density of states (DoS) of ZnMn_2_O_4_ with Mn vacancy defect. e) Comparative Ragone plot of the present system along with the previous works. f) An image for powering a digital watch using a single Zn/Mn‐d‐ZMO@C coin cell.

Therefore, to validate this, first‐principles calculations based on density functional theory^[^ ^66^ ^]^ were also performed to gain more insight into the electronic structure of the defect engineered ZnMn_2_O_4_ in comparison to the defect‐free ZnMn_2_O_4_ (see Note S2, Supporting Information). **Figure** **7**a–d shows the crystallographic structures of ZnMn_2_O_4_ and ZnMn_2_O_4_ with Mn vacancy defect along with their corresponding total density of states. It can be clearly seen that the defect‐free ZnMn_2_O_4_ exhibits semiconductor feature, while remarkably, the ZnMn_2_O_4_ with the Mn vacancy defect shows differently. Accordingly, it is worth noting that the Mn‐d‐ZMO@C sample showed a metallic behavior, which has a higher conductivity compared to the defect‐free ZnMn_2_O_4_. Thus, fast electron transport and an enhanced electrochemical property are expected. Therefore, the present in situ growth formation technique will open the new insight for fabricating advanced electrode materials with high performance.

Overall, it can be established that the carbon wrapped ZnO‐MnO electrode undergoes a “self‐oxidation” (Mn^2+^ to Mn^3+^) reactions initially and leads to the in situ formed of Mn‐d‐ZMO@C cathode. In situ PEIS and XRD, ex situ XRD, and XANES study revealed the development of the reproducible Zn/Mn‐d‐ZMO@C system. Moreover, these characterizations revealed that the reaction mechanism of the present Mn‐d‐ZMO@C cathode can be described by a combined intercalation–conversion–deposition reaction. The in situ generated Zn/Mn‐d‐ZMO@C battery exhibits excellent electrochemical performance. A long cycle with high capacity retention was achievable with the in situ formed cell (84% capacity maintained after 2000 cycles). A comparative study of the in situ grown Zn/Mn‐d‐ZMO@C cell with Zn/ZnMn_2_O_4_@C and Zn/ZnMn_2_O_4_‐MnO@C, respectively, identified that the Zn//Mn‐d‐ZMO@C cell exhibited improved electrochemical performance than that of the conventional cells, respectively. However, there are some issues to be addressed further. For example, the relatively lower Coulombic efficiency during the initial few cycles at low current density (≈100 mA g^−1^) is worth noting in Figure 4b. The deintercalation process, in general, is believed to be dominant at low current densities and, in that case, it is feasible that the deintercalated Zn^2+^ ions are not fully inserted back into the electrode bulk during the discharge reaction either due to the presence of irreversible sites or because of some hindrance to the motion of the intercalating Zn^2+^ ions. The low current density can motivate the formation of a relatively thick ZBS layer (compared to that at higher current densities) on the electrode surface during discharge cycling. This layer can act as a barrier to Zn‐intercalation thus making it difficult for the intercalation of all the deintercalated Zn^2+^ ions during charge cycling. Also, this irreversibility can be related to some unusual pH variation that can hinder the reversibility of either of the conversion reactions when cycled at low current densities. More studies are required to establish the exact reason for the irreversibility at low current densities. The charged Zn//Mn‐d‐ZMO@C cell could be used even 12 months later and able to use for long cycle with fewer capacity decay (86% capacity retention). It exhibited a promising energy density of 296 Wh kg^−1^ at 135 W kg^−1^ power density, more superior than these of earlier reported works on Zn/MnO_2_,^[^ ^67^ ^]^ Zn/Na_0.95_MnO_2_,^[^ ^68^ ^]^ Zn/ZnMn_2_O_4_‐Mn_2_O_3,_^[^ ^69^ ^]^ Zn/Mn_2_O_3,_^[^ ^70^ ^]^ ZnMn_2_O_4,_^[^ ^38^, ^71^, ^72^ ^]^ and Zn/MnO_2_.^[^ ^73^ ^]^ Moreover, the in situ formed Zn/Mn‐d‐ZMO@C cell can deliver 178 Wh kg^−1^ specific energy at a high specific power of 4050 W kg^−1^. A Clearer picture has been depicted in the Ragone plot (Figure 7e). As displayed in the Figure 7f, a single Zn/Mn‐d‐ZMO@C battery powers a digital watch, demonstrating the practical applicability of this battery.

## 3 Conclusions

In summary, ZnO‐MnO@C nanocomposite materials have been synthesized herein via the solvent dry process. An in situ Zn/Mn‐d‐ZMO@C cathode was formed in the Zn cell containing an aqueous 2 m ZnSO_4_ and 0.2 m MnSO_4_ electrolyte. The formation of ZnMn_2_O_4_ with the Mn vacancy along with Zn_4_(OH)_6_(SO_4_).*x*H_2_O phase was identified using in situ and ex situ XRD. The in situ formed Zn/Mn‐d‐ZMO@C cell delivered an initial discharge capacity of 194 mAh g^−1^ at 100 mA g^−1^ current rate. Moreover, it can retain 84% capacity over 2000 cycles. In situ and ex situ XRD proved the formation and reproducibility of the in situ formed Mn‐d‐ZMO@C electrode. In addition, in situ/ex situ X‐ray and electrochemical analyses revealed that a possible combined intercalation–conversion–deposition reaction governs the regulation mechanism in the present Mn‐d‐ZMO@C cathode. However, considering the existing debate in the regulation mechanism for manganese oxide cathodes of AZIBs, further studies are required to confirm the proposed reaction. Further, first‐principles calculations suggest higher conductivity of the Mn defected electrode compared to that of pure ZnMn_2_O_4_. The in situ formed Zn/Mn‐d‐ZMO@C cell can provide a high specific energy density of 296 Wh kg^−1^ at 135 W kg^−1^ specific power density. The charged in situ cell can also be cycled after being stored for a period of one year. This study opens new avenue for further development of cathode material for high performance and practical rechargeable AZIB system.

## 4 Experimental Section

ZnO‐MnO@C composite materials were simply synthesis from a solvent dry process. 70% HNO_3_, Zn(NO_3_)_2_.6H_2_O, Mn(NO_3_)_2_.4H_2_O from Sigma‐Aldrich and commercial acetylene black were used as a precursor. Initially, 2 m of Zn(NO_3_)_2_.6H_2_O and 4 m of Mn(NO_3_)_2_.4H_2_O were dissolved in 20 mL of concentrated HNO_3_ followed by the addition of 50 mg of acetylene black powder to the above solution under vigorous stirring. The solution was then dried at 80 °C in air. Finally, the dried powder was annealed at 400 °C, 500 °C, and 650 °C temperature separately for 4 h in an argon (Ar) atmosphere. Materials and electrochemical characterization are discussed in the Note S1 (Supporting Information).

## Conflict of Interest

The authors declare no conflict of interest.

## Supporting information

Supporting Information
