# Supplementary material for: In Situ Oriented Mn Deficient ZnMn2O4@C Nanoarchitecture for Durable Rechargeable Aqueous Zinc‐Ion Batteries
Source: Adv Sci (Weinh). 2021 Jan 4;8(4):2002636. doi: 10.1002/advs.202002636 (PMC7887583; doi:10.1002/advs.202002636)
Supplement: Supplementary file 1 — Supporting Information [file ADVS-8-2002636-s001.pdf]

WILEY-VCH

© 2020 Wiley-VCH GmbH

ADVANCED SCIENCE
Open Access

Supporting Information

for *Adv. Sci.,* DOI: 10.1002/advs.202002636

In Situ Oriented Mn Deficient $ZnMn_2O_4$@C Nanoarchitecture for Durable Rechargeable Aqueous Zinc-Ion Battery

Saiful Islam, Muhammad Hilmy Alfaruqi, Dimas Yunianto Putro, Sohyun Park, Seokhun Kim, Seulgi Lee, Mohammad Shamsuddin Ahmed, Vinod Mathew, Yang-Kook Sun, Jang-Yeon Hwang, and Jaekook Kim**

Supporting Information

In Situ Oriented Mn Deficient $ZnMn_2O_4$@C Nanoarchitecture for Durable Rechargeable Aqueous Zinc-Ion Battery

Saiful Islam,[a] Muhammad Hilmy Alfaruqi,[a,b] Dimas Yunianto Putro,[a] Sohyun Park,[a] Seokhun Kim,[a] Seulgi Lee,[a] Mohammad Shamsuddin Ahmed,[a] Vinod Mathew,[a] Yang-Kook Sun,[c] Jang-Yeon Hwang,[a,] and Jaekook Kim[a,]**

[a] Department of Materials Science and Engineering, Chonnam National University, Gwangju 500-757, South Korea
[b] Department of Metallurgical Engineering, Sumbawa University of Technology, Olat Maras, Sumbawa, West Nusa Tenggara, 84371, Indonesia
[c] Department of Energy Engineering, Hanyang University, Seoul 133-791, Republic of Korea

Supporting Information Note 1

X-ray diffraction was recorded using an X'Pert PANalytical Model High-resolution X-ray diffractometer with Cu Kα radiation (λ= 1.54056 Å). The XRD patterns were taken within the 2 θ range of 10 to 80 at 40 kV and 30 mA. The morphology of the sample ZnO-MnO was investigated by field-emission scanning electron microscopy (FE-SEM) and High-resolution transmission electron microscopy (HRTEM) techniques, recorded using S-4700 Hitachi model and Philips Tecnai F20 model operating at 200 keV, (KBSI, Chonnam Nation University) respectively. Raman spectra were recorded on a Raman microscope with an excitation laser beam wavelength of 532 nm with laser power of 1.3 mW. X-ray photoelectron spectroscopy (XPS) was measured using a Multilab 2000 model (Thermo Scientific Instrument). Al Kα was used as an X-ray source and the spectrometer was calibrated with respect to the C 1s peak binding energy of 284.6 eV. *In situ* and *ex-situ* XRD measurement was done using synchrotron beamline at 1D KIST-PAL (Pohang Accelerated Laboratory). High energy 2.5G eV with 200 mA current was employed to record XRD patterns at 0.692649 Å wavelength and the obtained data were managed using Fit2D software and finally re-plotted with respect to the λ-value of 1.5414 Å. Furthermore, synchrotron X-ray absorption

spectroscopy (XAS) technique at the BL7D beamline of Pohang light Source (PLS) in high energy 2.5 GeV with around 200 mA current condition was used to measure the ex-situ ZnO-MnO@C sample. The ATHENA software was used to analyse the collected XAS data.

The ZnO-MnO@C, $ZnMn_2O_4$@C and $ZnMn_2O_4$-MnO@C electrode was prepared by mixing active materials (ZnO-MnO@C), Ketchjen black (KB) and Teflonated acetylene black (TAB) in the ratio 7:1:1 and pasted onto stainless steel. The past was dried for 12h in 120 ˚C pre-heated oven. A 2032-type coin cell was assembled by placing glass fiber between Zn metal anode and cathode. An aqueous solution of 2 M $ZnSO_4$ and 0.2 M $MnSO_4$ was used as an electrolyte. A BTS 2004H model (NAGANO) battery tester was used to check the galvanostatic charge-discharge in different conditions. The potential window was fixed from 1.9 to 0.8 V Vs. Zn/Zn^{2+}. Cyclic voltammetry (CV) and in-situ preliminary electrochemical impedance spectroscopy (PEIS) analysis was performed using AUTOLAB PGSTAT302N.

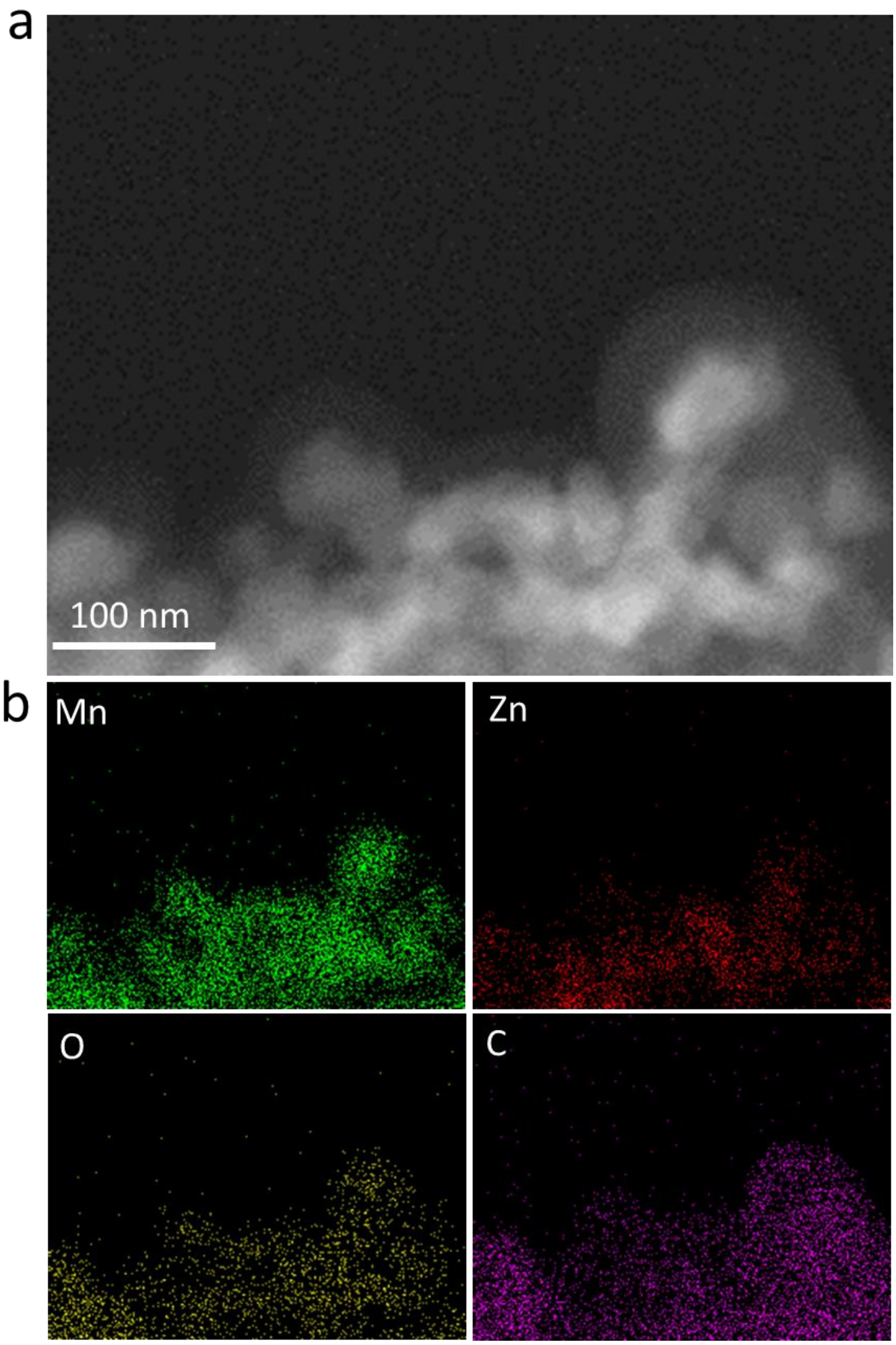

Figure S1: (a) The HAADF-TEM image and (b) elemental mapping images of ZnO-MnO@C sample.

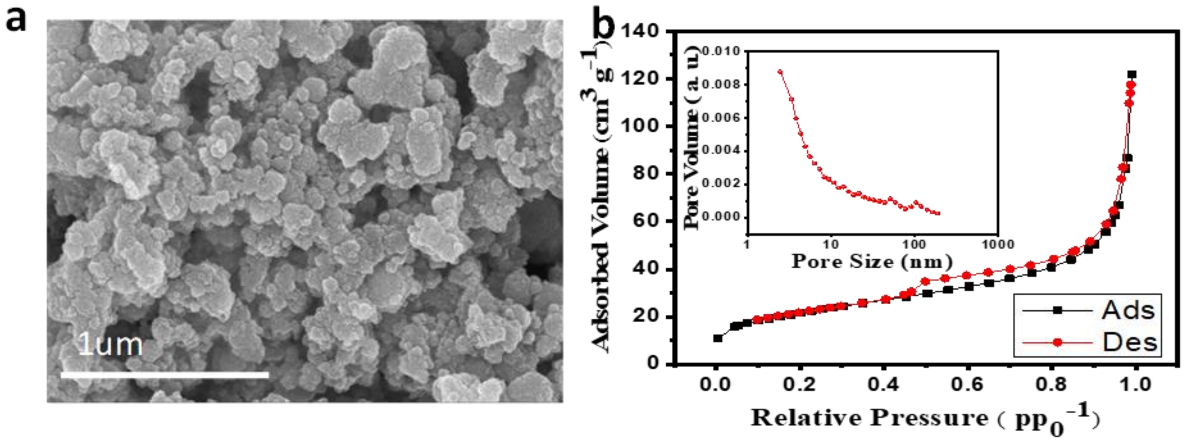

Figure S2: (a) The SEM image and (b) N_2-adsorption desorption isotherm of ZnO-MnO@C powder. Inset: the pore distribution of corresponding sample.

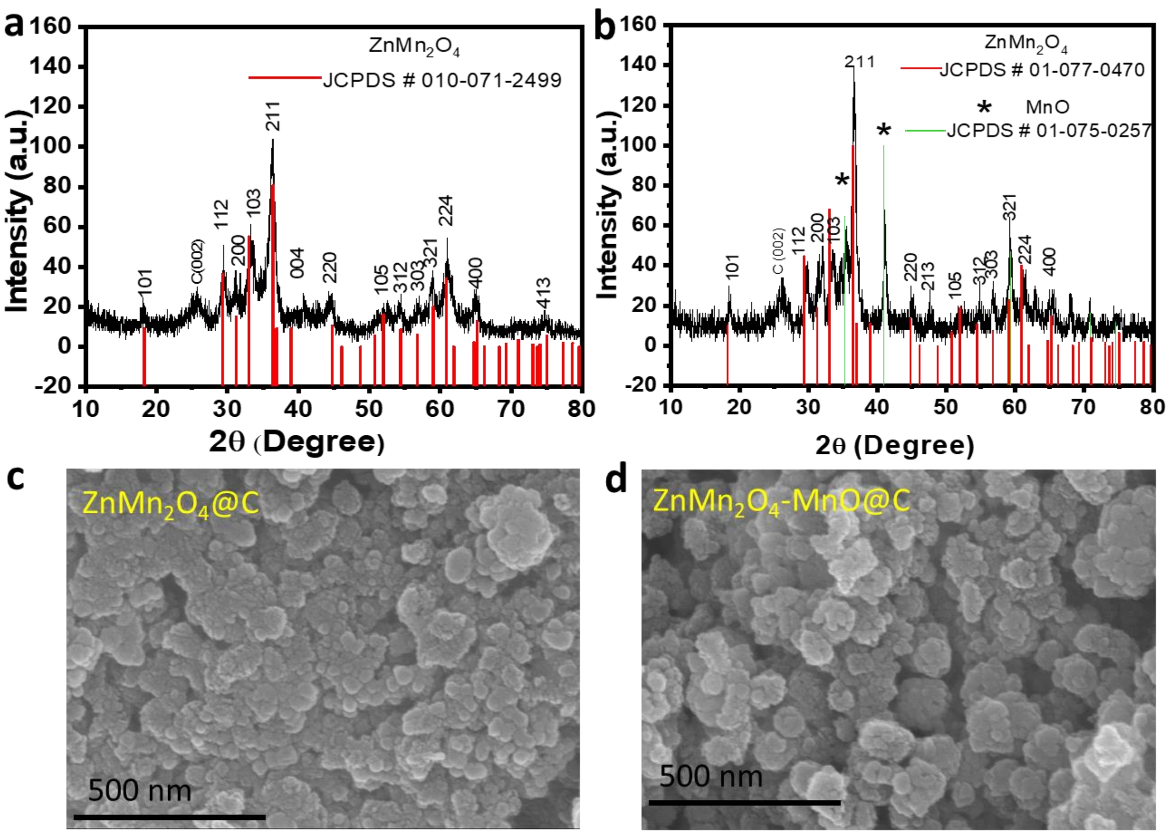

Figure S3: XRD pattern of (a) $ZnMn_2O_4$@C and (b) $ZnMn_2O_4$-MnO@C and SEM images of (c) $ZnMn_2O_4$@C and (d) $ZnMn_2O_4$-MnO@C.

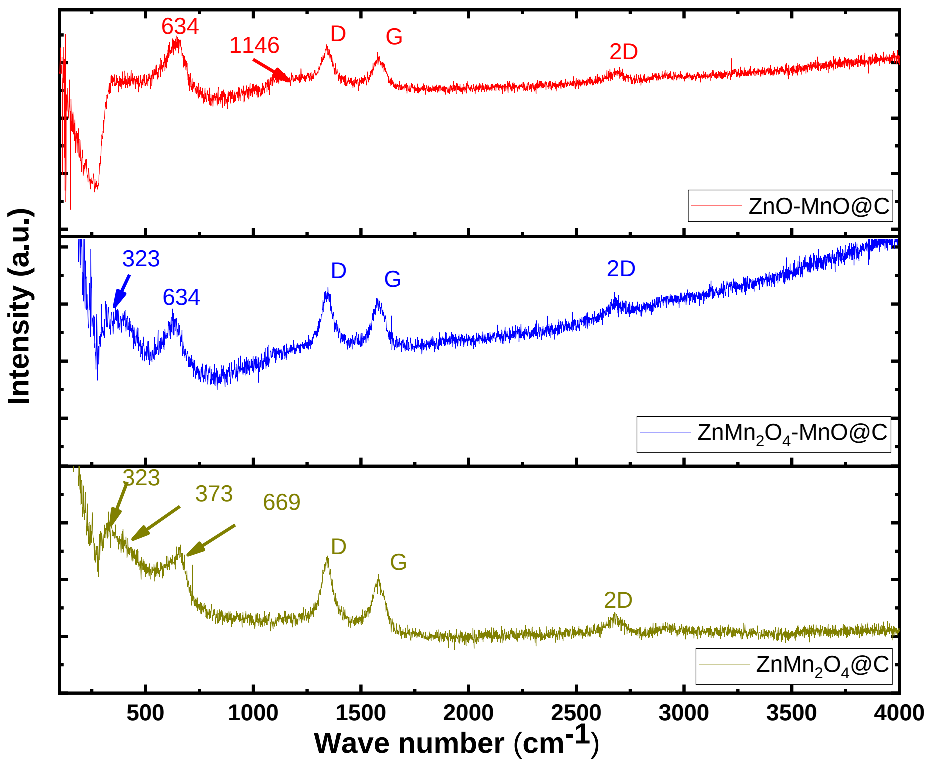

Figure S4: Raman spectra for carbon coated $ZnMn_2O_4$, $ZnMn_2O_4$-MnO and ZnO-MnO.

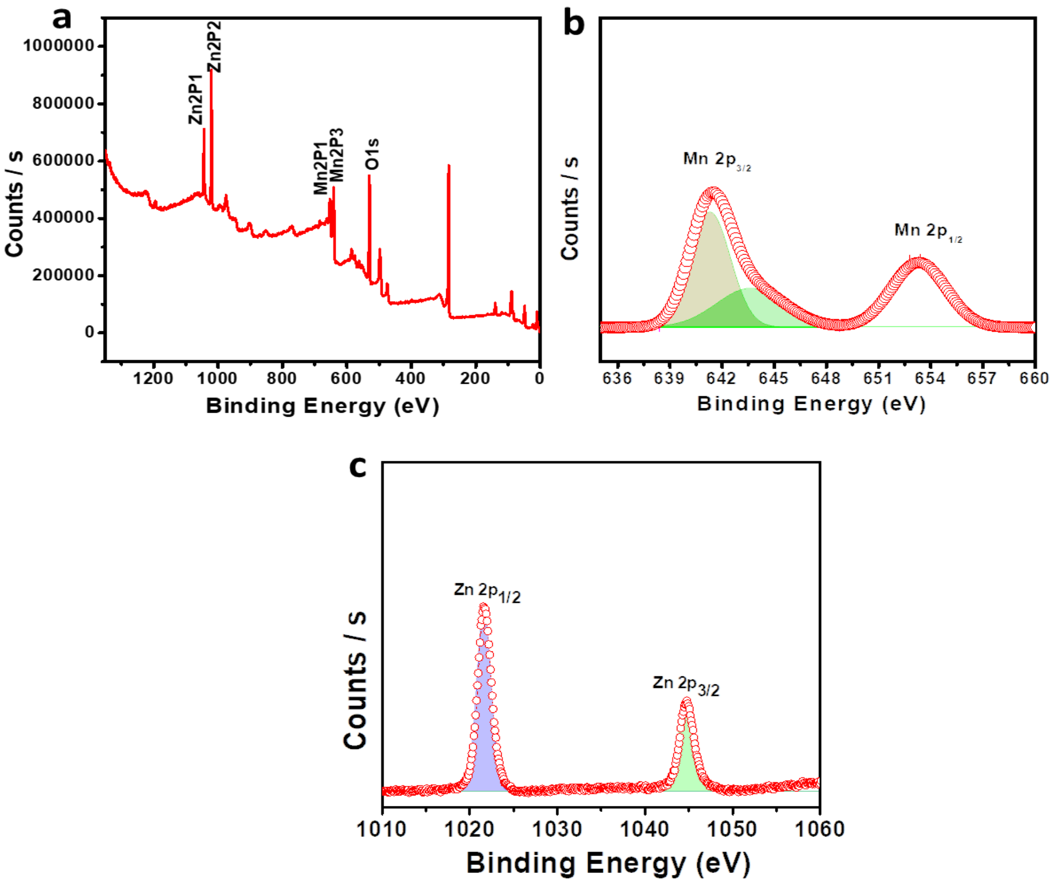

Figure S5: (a) Survey curve of XPS for ZnO-MnO@C powder core level of XPS (b) Mn2p and (c) Zn2p spectra of ZnO-MnO@C sample.

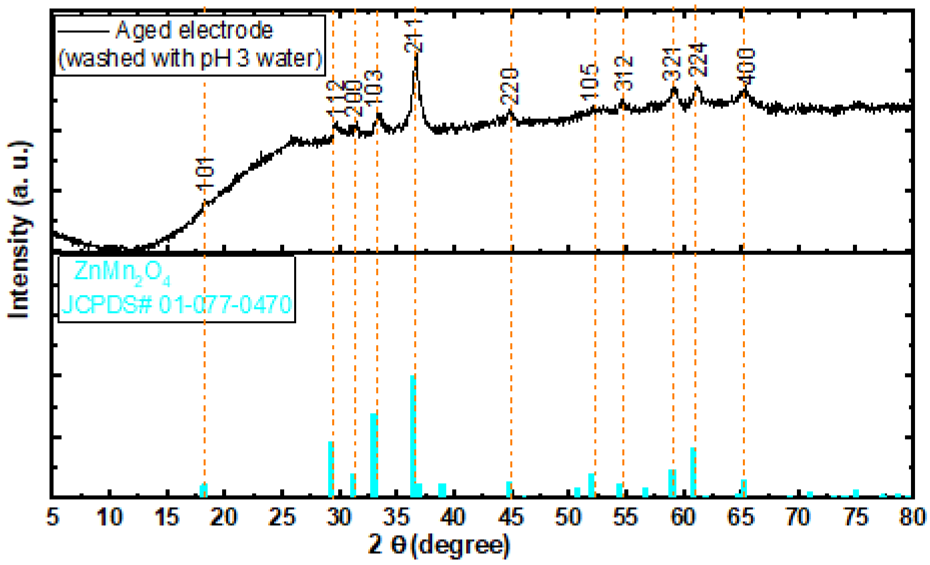

Figure S6: XRD pattern of aged ZnO-MnO@C electrode after washed with pH 3 water.

Table S1: ICP results of the ZnO-MnO@C powder, aged ZnO-MnO@C electrode and 2M $ZnSO_4$ electrolyte after the immersion of ZnO-MnO@C electrode.

Sample	Element	Concentration (ppm)
ZnO-MnO@C	Mn	53000
	Zn	26800
Aged ZnO-MnO@C electrode (in 2M $ZnSO_4$)	Mn	50100
	Zn	30300
Electrolyte (2M $ZnSO_4$) after aging the electrode for 24 h	Mn	111
	Zn	103000
1st charge	Mn	65200
	Zn	16000

As depicted in **Table S1** the Mn to Zn ratio was measured to be 1.98 and 1.65 for the ZnO-MnO@C powder and aged ZnO-MnO@C electrode, respectively. That indicates Mn dissolution occurred during aging process.

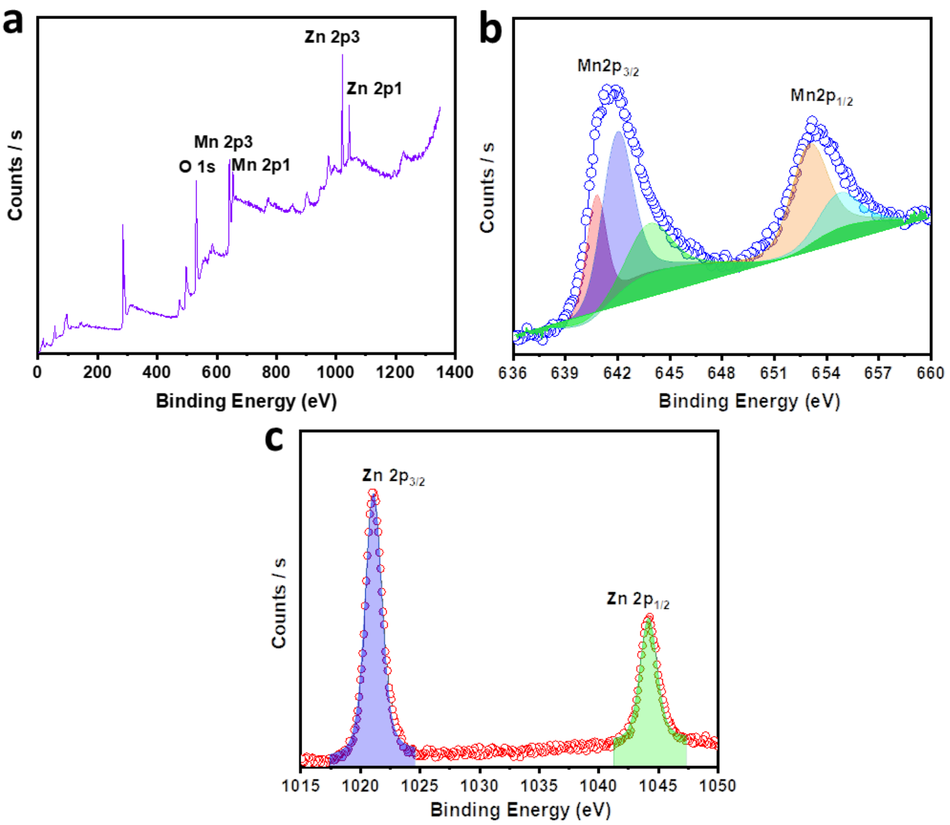

Figure S7: (a) XPS survey scan of the aged ZnO-MnO@C electrode. (b & c) XPS for Mn and Zn element of aged ZnO-MnO@C sample.

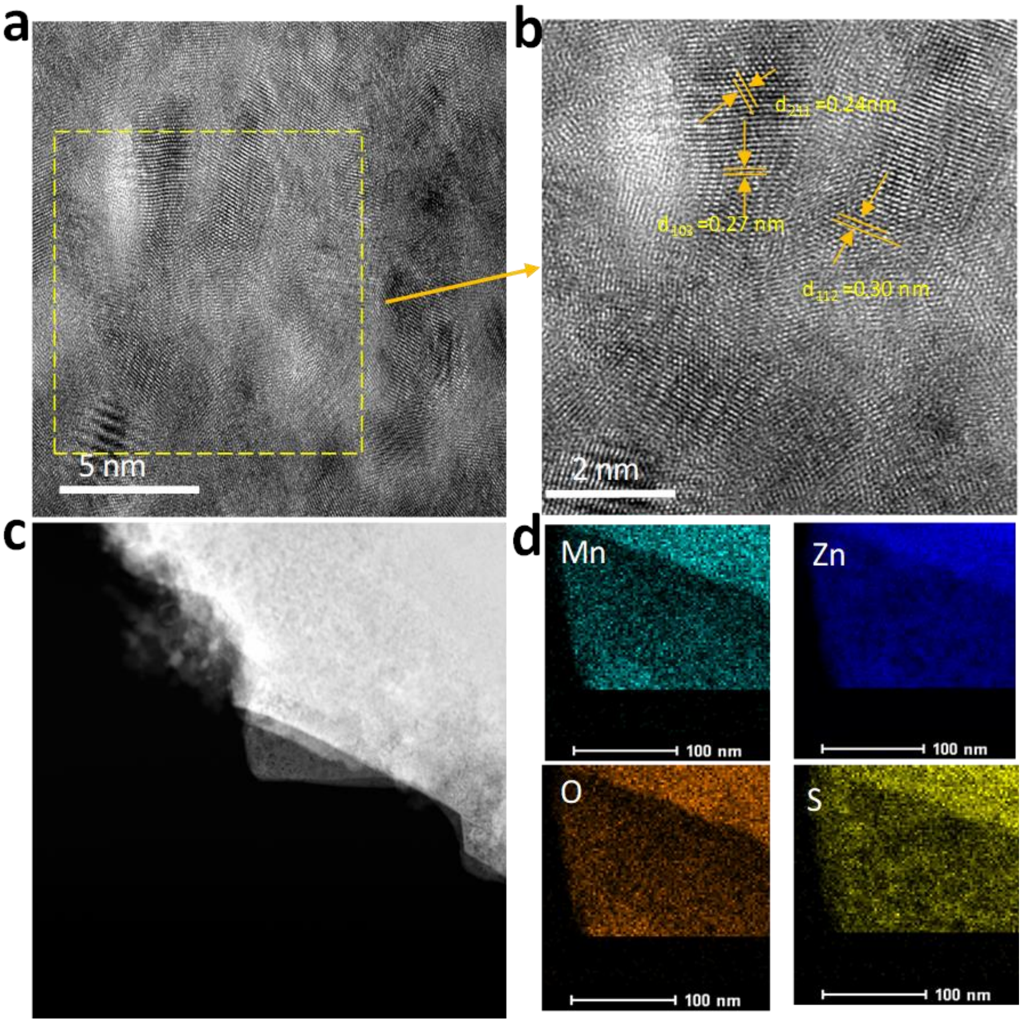

Figure S8: HRTEM images of aged ZnO-MnO@C electrode (a) at 5 nm scale and (b) at 2 nm showing lattice plane of $ZnMn_2O_4$. (c) HAADF-STEM image and (d) Elemental mapping.

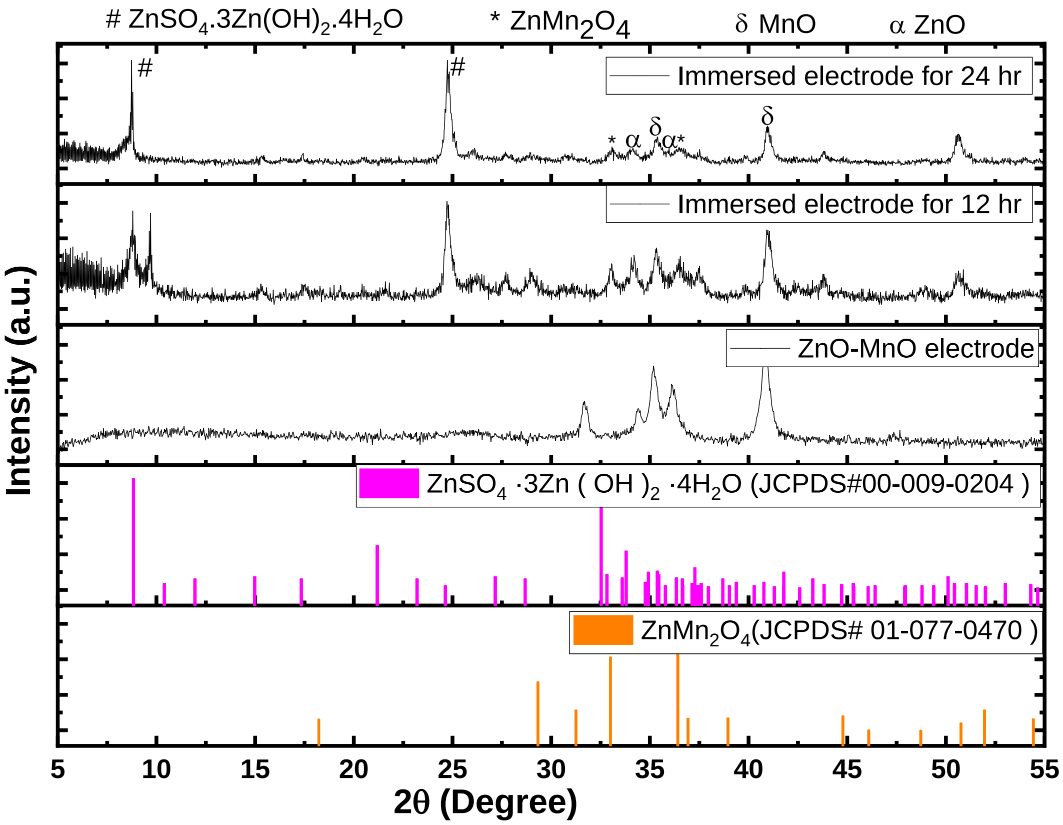

Figure S9: *Ex situ* XRD of the ZnO-MnO@C electrode after immersion in 5 ml aqueous solution of 2 M $ZnSO_4$ and 0.2 M $MnSO_4$.

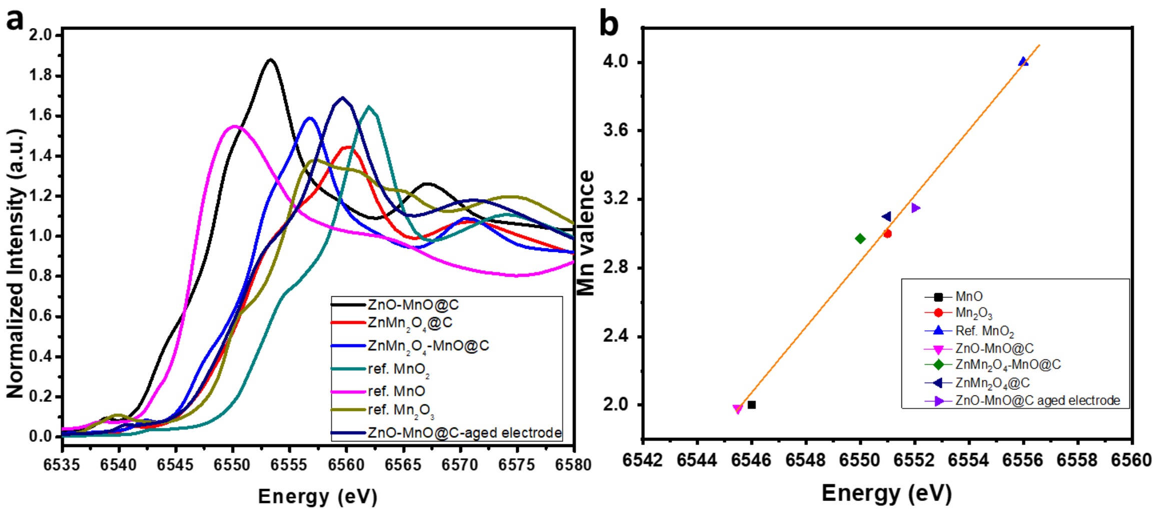

Figure S10: (a) XANES for carbon enfolded $ZnMn_2O_4$, $ZnMn_2O_4$-MnO, ZnO-MnO, and aged ZnO-MnO electrode with reference materials MnO, MnO_2, Mn_2O_3. And (b) Respective Mn valence versus energy curve.

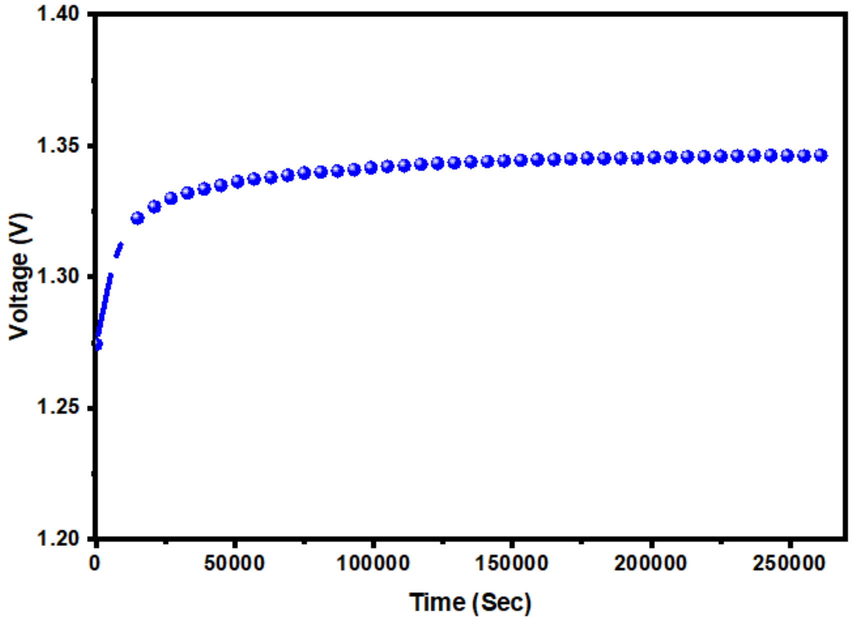

Figure S11: Voltage variation plot of the Zn/ZnO-MnO@C cell during aging process.

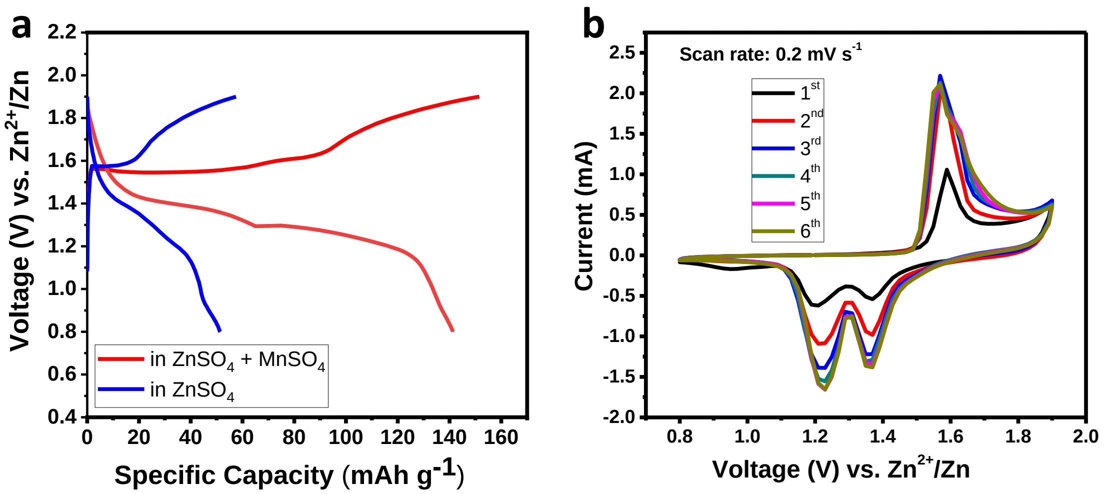

Figure S12: (a) Charge-discharge curve of Zn/ZnO-MnO@C in 2 M $ZnSO_4$ with and without 0.2 M $MnSO_4$ at 300 mA g^{-1} current rate. (b) First few CV cycle of Zn/ZnO-MnO@C in additive containing electrolyte at a scan rate of 0.2 mV s^{-1}.

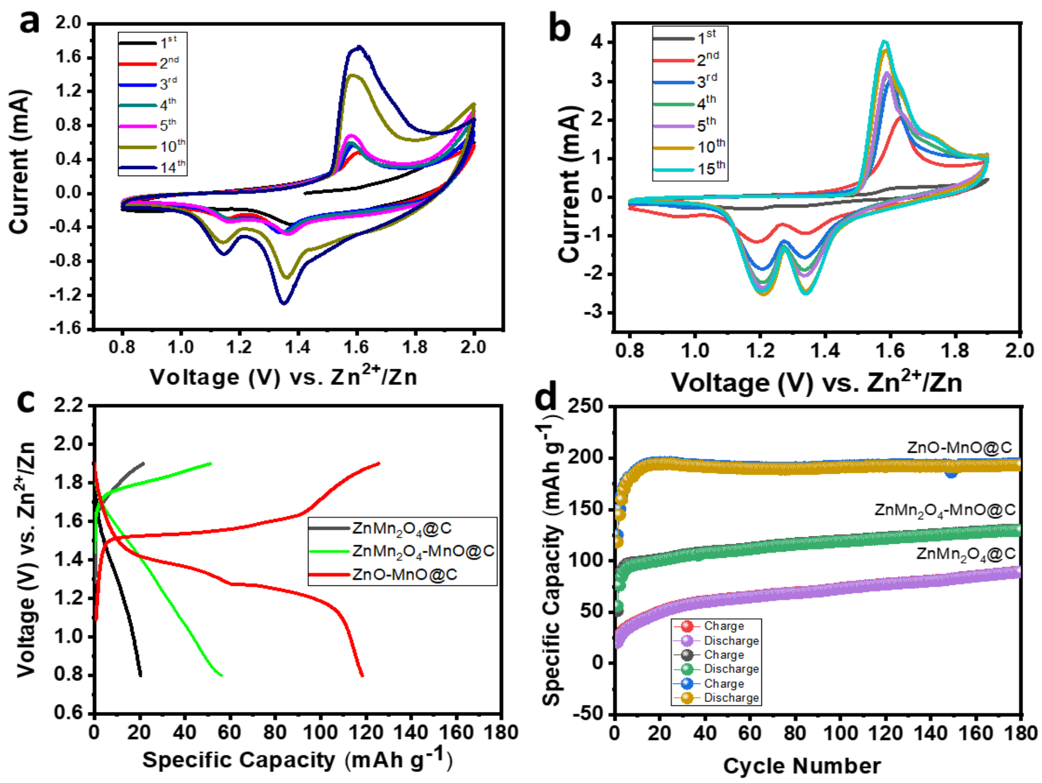

Figure S13: Comparative electrochemical properties of $ZnMn_2O_4$@C, $ZnMn_2O_4$-MnO@C and ZnO-MnO@C. Cyclic voltammetry plots of (a) $ZnMn_2O_4$@C and (b) $ZnMn_2O_4$-MnO@C at 0.2 mV s^{-1} scan rate. (c) ECD profiles and (d) cycle performance of $ZnMn_2O_4$@C, $ZnMn_2O_4$-MnO@C and ZnO-MnO@C at 500 mA g^{-1} current density.

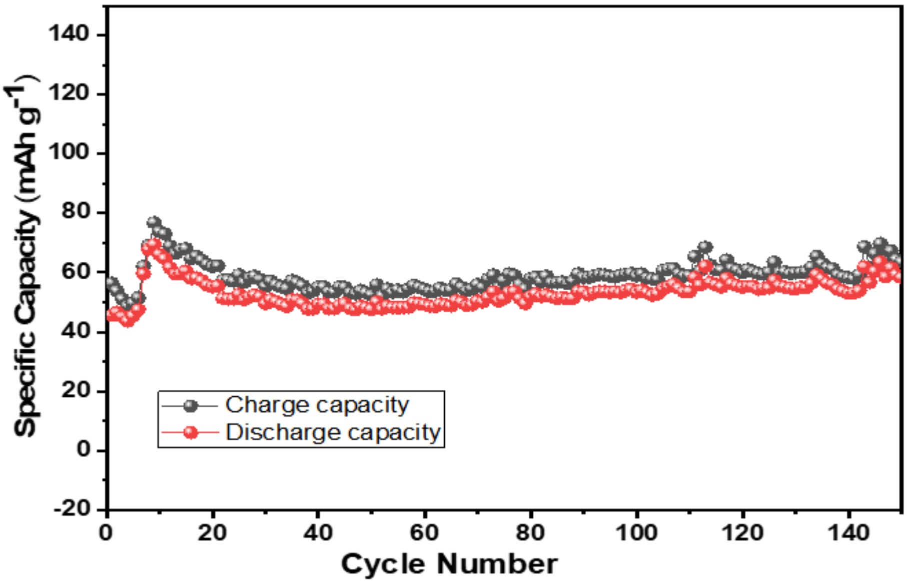

Figure S14: Cycle ability of one-year old Zn/ZnO-MnO@C cell at 100 mA g^{-1} current density.

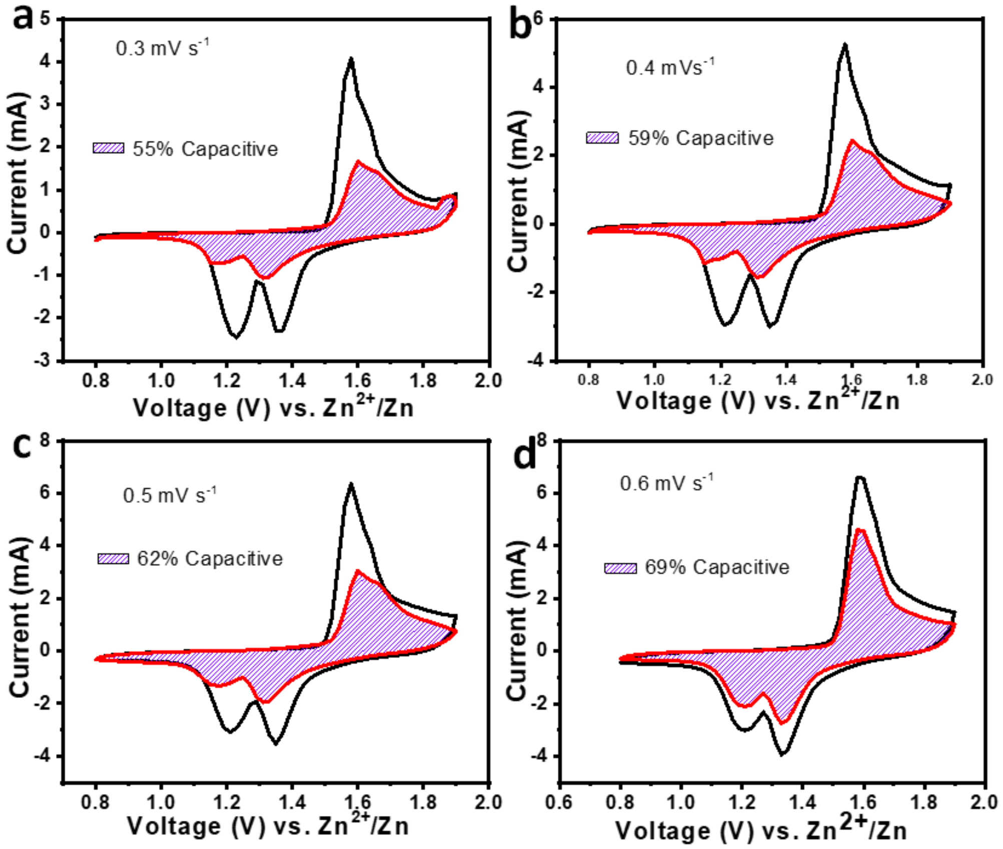

Figure S15: Capacitive contribution of ZnO-MnO@C sample at different scan rates.

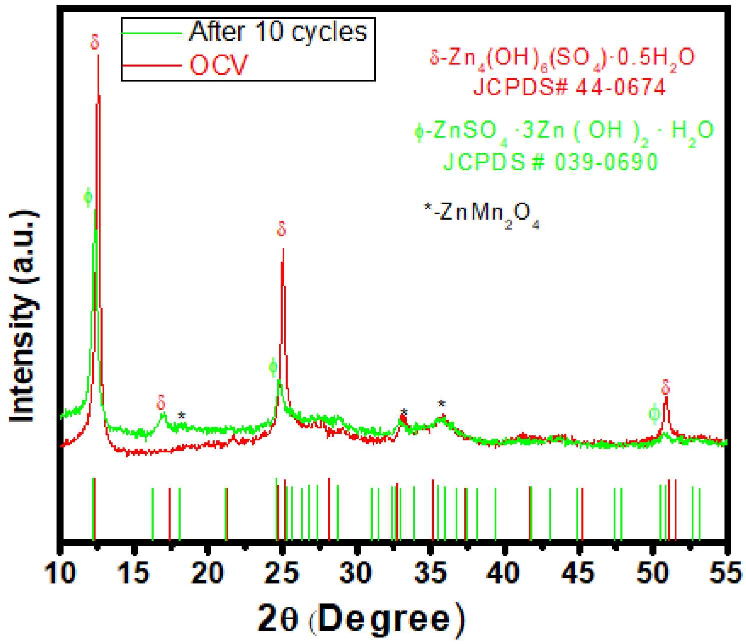

Figure S16: *Ex situ* XRD of ZnO-MnO@C electrode at OCV state and after 10 discharge cycle.

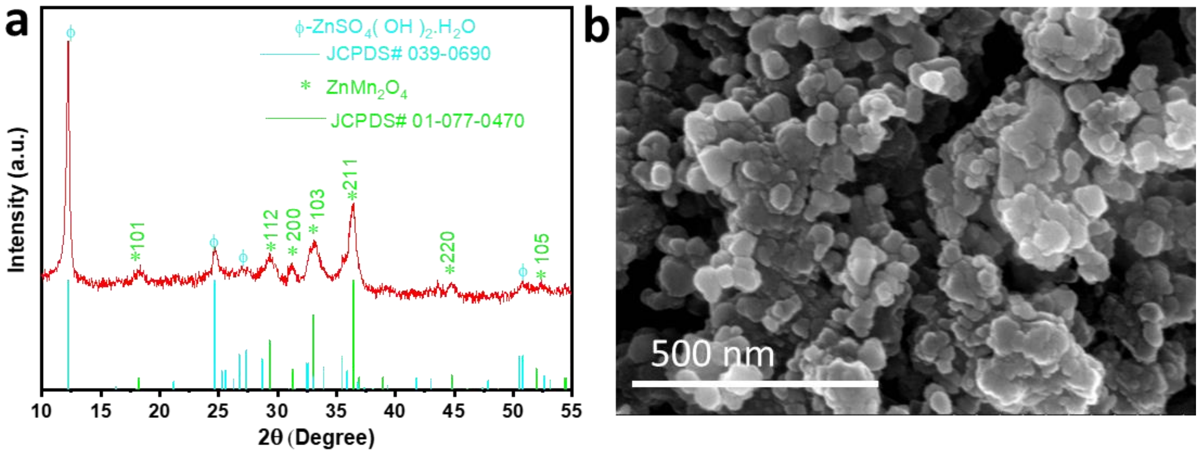

Figure S17: (a) XRD pattern and (b) SEM image of the *in situ* formed Mn-d-$ZnMn_2O_4$@C electrode after 20 discharge cycles.

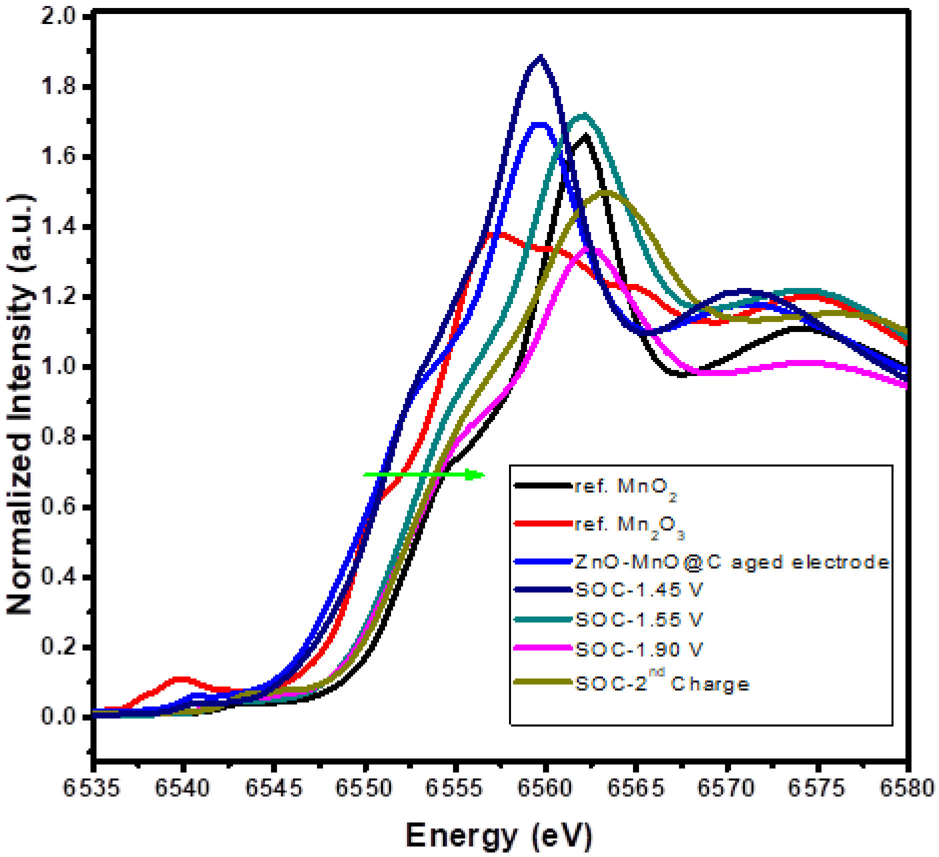

Figure S18: *Ex situ* XANES of Zn/ZnO-MnO@C system at different state of charge.

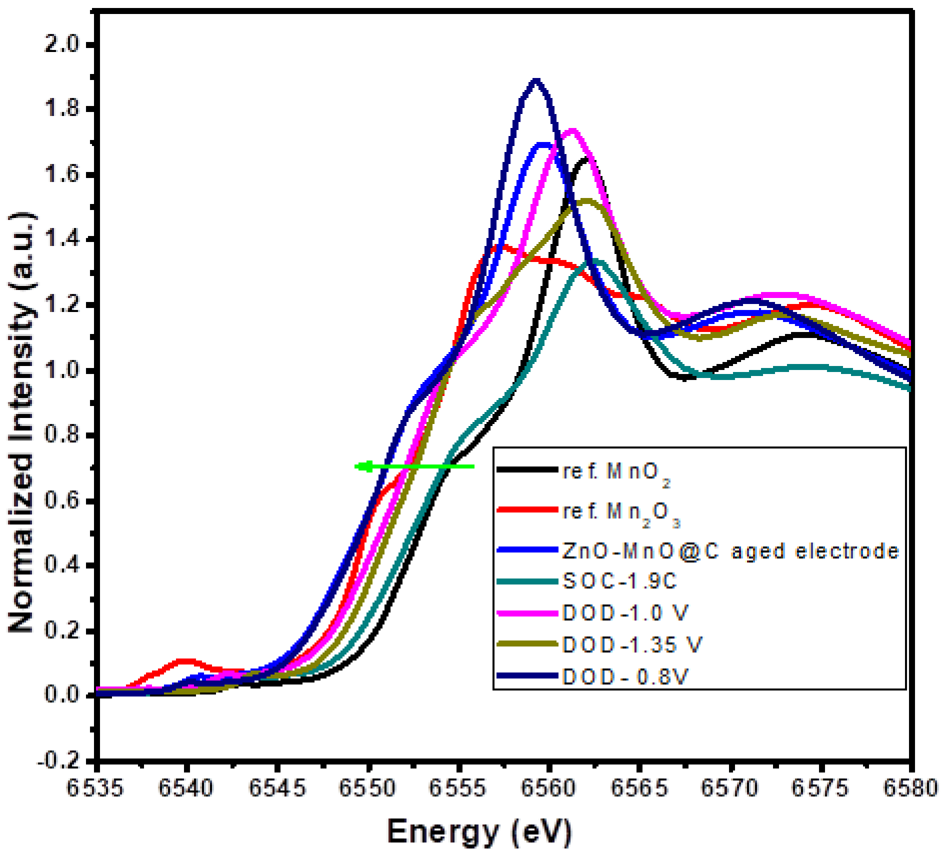

Figure S19: *Ex situ* XANES of Zn/ZnO-MnO@C system at different depth of discharge.

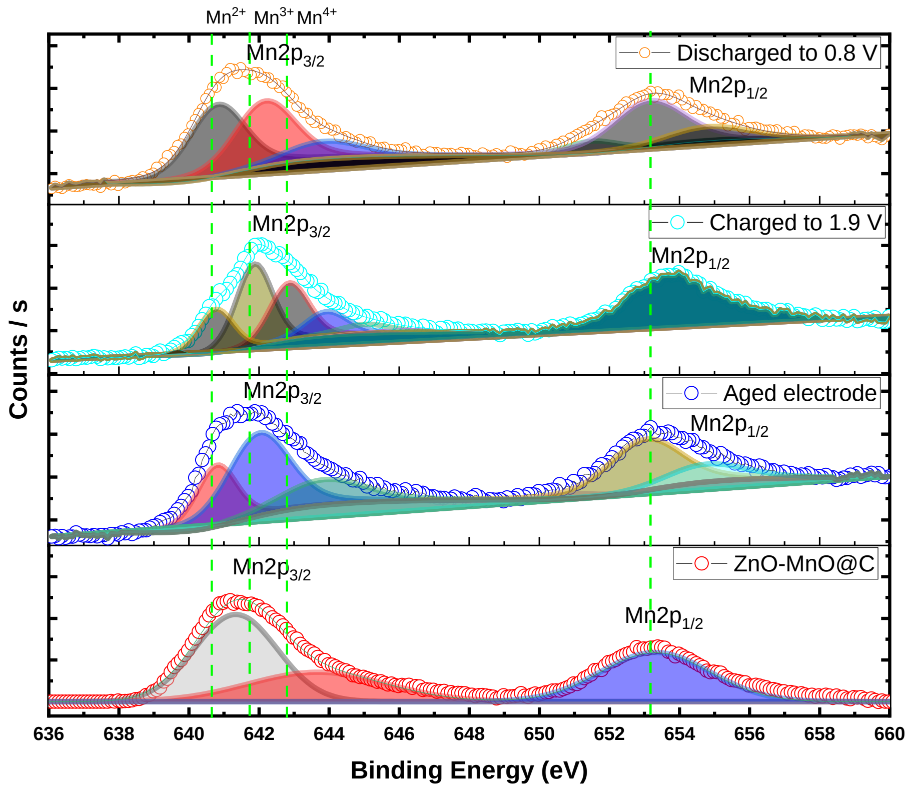

Figure S20: *Ex situ* XPS spectra of the fresh, aged and cycled ZnO-MnO@C electrode.

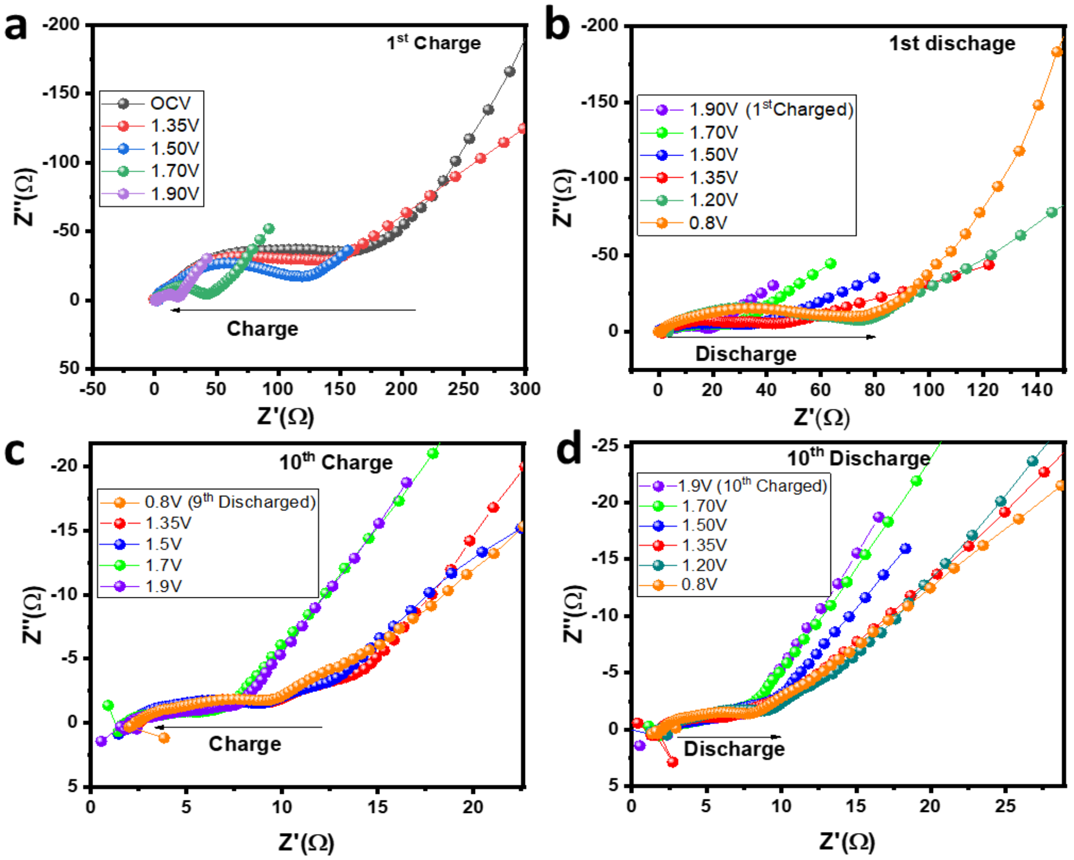

Figure S21: *In situ* PEIS of Zn/ZnO-MnO@C at different cycles: (a &b) 1st cycle and (c & d) 10th cycle.

Supporting Information Note 2

Computational Methods

In this study, first-principles calculations based on density functional theory (DFT) were performed using Quantum-Espresso program with projector augmented wave (PAW) pseudopotential and Perdew-Burke-Emzerhof (PBE) exchange-correlation functional.[1,2] A plane-wave basis set with a cutoff energy of 30 Ry (408 eV) was used. The lattice parameters and positions of atoms in the defect-free and Mn vacancy defect $ZnMn_2O_4$ structures were relaxed using Broyden-Fletcher-Goldfarb-Shanno (BFGS) and the Brillouin zones were sampled using a k-point mesh of 4 × 4 × 2. In addition, for density of states (DOS) calculations, DFT+U method was applied with on-site potential *U* of 3.9 eV for manganese and a k-point mesh of 2 × 2 × 1 was used.[3]

References:

[1] P. Giannozzi, O. Andreussi, T. Brumme, O. Bunau, M. Buongiorno Nardelli, M. Calandra, R. Car, C. Cavazzoni, D. Ceresoli, M. Cococcioni, N. Colonna, I. Carnimeo, A. Dal Corso, S. De Gironcoli, P. Delugas, R. A. Distasio, A. Ferretti, A. Floris, G. Fratesi, G. Fugallo, R. Gebauer, U. Gerstmann, F. Giustino, T. Gorni, J. Jia, M. Kawamura, H. Y. Ko, A. Kokalj, E. Kücükbenli, M. Lazzeri, M. Marsili, N. Marzari, F. Mauri, N. L. Nguyen, H. V. Nguyen, A. Otero-De-La-Roza, L. Paulatto, S. Poncé, D. Rocca, R. Sabatini, B. Santra, M. Schlipf, A. P. Seitsonen, A. Smogunov, I. Timrov, T. Thonhauser, P. Umari, N. Vast, X. Wu, S. Baroni, *J. Phys. Condens. Matter* **2017,** *29*,46.

[2] J. P. Perdew, K. Burke, M. Ernzerhof, *Phys. Rev. Lett.***1997**, *8730*, 46920.

[3] A. Jain, G. Hautier, C. J. Moore, S. P. Ong, C. C. Fischer, T. Mueller, K. A. Persson, G. Ceder, *Comput. Mater. Sci.* **2011,** *50*, 2295-2310.
